# Green Recovery of Bioactive Compounds from Bergamot (*Citrus bergamia*) By-Products: Sustainable Extraction, Food Applications, and Health-Promoting Properties

**DOI:** 10.3390/foods15111955

**Published:** 2026-06-01

**Authors:** Alessandra De Bruno, Antonio Gattuso, Gianluca Tripodi, Mauro Lombardo, Sara Baldelli, Gilda Aiello

**Affiliations:** 1 Department of Human Sciences and Promotion of the Quality of Life, San Raffaele University, 00166 Rome, Italy; alessandra.debruno@uniroma5.it (A.D.B.); mauro.lombardo@uniroma5.it (M.L.); sara.baldelli@uniroma5.it (S.B.); gilda.aiello@uniroma5.it (G.A.); 2 Department of AGRARIA, University Mediterranea of Reggio Calabria, 89124 Reggio Calabria, Italy; 3 IRCCS San Raffaele Roma, 00166 Rome, Italy

**Keywords:** bergamot by-products, circular bioeconomy, citrus bergamia, food waste valorisation, functional foods, natural deep eutectic solvents (NADES), polyphenols

## Abstract

Bergamot (*Citrus bergamia Risso* et Poiteau), a citrus fruit typically cultivated in the Mediterranean basin, represents a valuable source of bioactive compounds, including polyphenols, flavanone glycosides, and essential oil constituents, which are associated with antioxidant and metabolic effects. Notably, these compounds are highly concentrated not only in the edible fraction but also in industrial by-products, such as peel and pomace, which represent an underexploited resource for sustainable valorisation. This review examines recent advances (2020–2025) in the recovery and application of bioactive compounds from bergamot by-products (BBP), with a focus on green extraction technologies, including ultrasound- and microwave-assisted extraction, enzyme-assisted processes, supercritical fluids, and natural deep eutectic solvents. Particular attention is given to the incorporation of BBP-derived extracts into food systems as natural antioxidants, flavouring agents, and functional ingredients. In addition, current evidence on their nutritional relevance and biological activities, including antioxidants, anti-inflammatory, and lipid-lowering effects, is critically discussed. The integration of green extraction technologies with stabilization strategies, such as microencapsulation, supports the development of clean-label foods enriched with bergamot bioactives, contributing to both product functionality and sustainability. Overall, bergamot by-products (BBP) emerged as a promising model for the circular recovery of natural bioactive compounds and their incorporation into innovative functional food formulations.

## 1. Introduction

The agri-food industry is increasingly required to adopt sustainable processing strategies to reduce waste, minimize environmental impact, and produce natural ingredients suitable for clean-label food products. In this context, the transition toward a circular bioeconomy has become a key objective, promoting the valorisation of agro-industrial residues as sources of value-added functional compounds [1,2,3].

Among agro-industrial residues, citrus by-products represent a quantitatively relevant waste stream. In bergamot industrial processing, residues may account for approximately 40–50% of the fresh fruit mass and mainly consist of peel, pulp residues, seeds, and pomace, while simultaneously constituting chemically rich matrices for the recovery of bioactive compounds such as polyphenols, dietary fibres, and limonoids. These materials have attracted increasing attention for their potential application as functional food ingredients and natural antioxidants [4,5,6].

Bergamot (*Citrus bergamia* Risso et Poiteau), a citrus species predominantly cultivated in the Reggio Calabria province (Southern Italy), although limited cultivation also occurs in other Mediterranean areas. It is widely recognized for its essential oil, obtained by cold pressing the peel and widely used in high-quality perfumery and cosmetics, and other industrial sectors. The extraction process generates substantial quantities of residual biomass, which remains largely underutilized despite its rich bioactive composition.

In addition to its essential oil, bergamot is a relevant source of polyphenols and flavonoids, including unique C-glycosyl flavonoids such as brutieridin and melitidin [7], which have been associated with cardiometabolic, antioxidant, and anti-inflammatory effects [8,9,10,11].

Notably, these compounds are largely retained in processing by-products, highlighting their technological and functional potential.

Despite the increasing scientific interest in bergamot phytochemicals and their biological activities, the valorisation of bergamot pomace as a functional food ingredient remains relatively underexplored. Currently, most industrial residues are mainly destined for livestock feed or bioenergy production, despite evidence showing that bergamot pomace and peel-derived fractions exhibit high phenolic content and strong antioxidant activity, as assessed by commonly used spectrophotometric assays such as DPPH, ABTS, and FRAP [12,13,14].

Recent research has focused on the development of green extraction technologies aimed at improving recovery efficiency while reducing solvent use, energy consumption, and processing time. Techniques such as ultrasound- and microwave-assisted extraction, enzyme-assisted processes, supercritical fluids, and natural deep eutectic solvents have emerged as promising approaches for obtaining food-grade extracts [15,16,17,18].

In parallel, growing attention has been devoted to the incorporation of BBP into food systems, including flours, extracts, dried fractions, and microencapsulated formulations, although technological challenges remain, particularly regarding compound stability, sensory impact, and bioavailability [14,19,20,21].

Despite these advances, previous reviews have mainly addressed the biological, anti-inflammatory, pharmacological, and nutraceutical properties of bergamot extracts, essential oil, and by-products, whereas less attention has been devoted to their technological and food-oriented valorisation. Therefore, the literature still lacks an integrated framework linking sustainable recovery, stabilization strategies, formulation challenges, food applications and industrial applicability of BBP.

Based on this gap, the present review provides an integrated overview of recent developments (2020–2025) in green extraction strategies, stabilization approaches, and food applications of bergamot-derived bioactive compounds. Particular attention is devoted to their potential role as functional ingredients for sustainable food innovation within a circular bioeconomy perspective.

**Figure 1:**
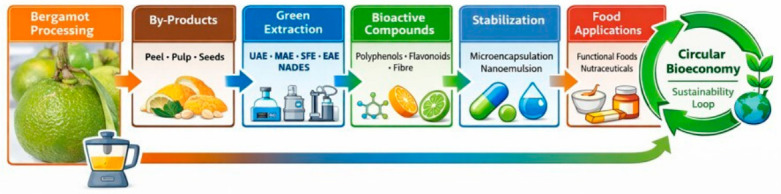
Integrated valorisation pathway of bergamot by-products. Abbreviations: UAE, ultrasound-assisted extraction; MAE, microwave-assisted extraction; SFE, supercritical fluid extraction; EAE, enzyme-assisted extraction; NADES, natural deep eutectic solvents.

To provide a visual overview of the conceptual framework of this review, Figure 1 summarizes the integrated valorisation pathway of BBP, from processing residues and green extraction technologies to bioactive compound recovery, stabilization strategies, food applications, and circular bioeconomy perspectives.

### Literature Search Strategy

The literature included in this review was identified through a structured search conducted in Scopus, Web of Science, and PubMed. The search was performed using combinations of the following keywords: *Citrus bergamia*; bergamot; by-products; pomace; polyphenols; bioactive compounds; green extraction; natural deep eutectic solvents; functional foods; and antioxidant activity. Boolean operators were used to combine terms related to the plant matrix, target compounds, extraction technologies, stabilization strategies, food applications, and biological properties.

The search focused on articles published between 2020 and 2025 to capture recent advances in the sustainable recovery, characterization, and application of bioactive compounds from BBP. Earlier studies were included only when they provided essential background information, methodological support, or relevant comparative evidence. Only peer-reviewed full-text articles written in English were considered.

Studies were selected based on their relevance to the objectives of this review. In particular, eligible studies included those that: (a) characterize the chemical or phytochemical composition of BBP; (b) describe extraction, purification, or stabilization techniques for bergamot-derived bioactive compounds; (c) investigate the incorporation of bergamot extracts or fractions into food systems; (d) evaluate biological activities through in vitro, in vivo, or clinical studies relevant to food or nutraceutical applications; and (e) assess bioaccessibility or bioavailability, safety, or technological functionality.

Articles were excluded if they were not peer-reviewed, lacked full-text availability, were not written in English, or were not relevant to the scope of this review. Duplicate records were removed during the screening process.

## 2. Chemical Composition and Bioactive Profile of Bergamot By-Products

The phytochemical composition of BBP, namely peel (flavedo and albedo), pomace and seeds, is exceptionally complex and represents an underexploited resource characterized by a diverse array of secondary metabolites, including polyphenols, flavonoids, phenolic acids, limonoids, coumarins, alkaloids, and volatile terpenoids.

From a circular economy perspective, a detailed understanding of the phytochemical profile of BBP is essential for the rational design of green extraction strategies, stabilization approaches, and food applications.

Notably, BBP are characterized by a strong enrichment in phenolic compounds and structural polysaccharides, which makes them especially suitable for green extraction and valorisation processes. The physicochemical properties of these matrices, such as high fibre content, presence of bound phenolics, and retention of volatile compounds, strongly influence extractability, stability, and potential applications as functional food ingredients. Therefore, a detailed understanding of their phytochemical profile is essential for optimizing recovery strategies and maximizing their industrial exploitation. Table 1 summarizes the main BBP, their origin, and their major bioactive compounds.

### 2.1. Polyphenols and Flavonoids

Polyphenols constitute the most abundant and biologically relevant class of secondary metabolites in *Citrus bergamia*, particularly in peel, albedo/flavedo, pomace and leaves-derived fractions. The polyphenolic profile of bergamot is dominated by flavanone glycosides, such as naringin, neohesperidin, neoeriocitrin, and eriocitrin, which are unevenly distributed across fruit tissues and are highly concentrated in the flavedo and albedo layers. Therefore, these compounds are present in high concentrations in pomace after juice and essential oil processing, contributing to the strong antioxidant potential of [10,12].

Structurally, the main bergamot flavonoids include flavanone O-glycosides derived from naringenin, hesperetin, and eriodictyol aglycones, as well as distinctive O-linked 3-hydroxy-3-methylglutaryl (HMG)-flavanones such as brutieridin and melitidin. These compounds differ in their glycosylation patterns, substituent groups, and polarity, which influence their solubility, extractability, and behaviour in aqueous or green solvent systems. Among the major flavanone glycosides, neoeriocitrin, naringin, and neohesperidin are widely reported in peel, pomace, and bergamot juice (BJ).

Brutieridin and melitidin should be considered separately as characteristic bergamot HMG-flavanones. They possess an HMG ester moiety and have been associated with lipid-lowering activity through interaction with HMG-CoA reductase.

Importantly, brutieridin and melitidin are predominantly localized in peel and albedo tissues, making bergamot pomace a strategic raw material for their selective recovery. High-performance liquid chromatography (HPLC) and LC-MS studies confirm that the Bergamot Polyphenolic Fraction (BPF) contains more than 60 individual flavonoid and phenolic derivatives, including trace amounts of diosmin, luteolin-7-O-glucoside, apigenin, and rutin [13]. Chromatographic profiling of the BPF derived from fruit juice, shows that the concentration of individual flavonoids expressed as naringin equivalents (mg per g of extract) can be substantial. For example, chromatographic profiling of BPF reported neoeriocitrin, naringin, neohesperidin, melitidin, and brutieridin at approximately 94.7, 38.0, 20.3, 22.8, and 30.2 mg/g of extract, respectively, with neoeriocitrin representing the most abundant compound [10].

Total polyphenolic content (TPC) measured through spectrophotometric assays such as Folin–Ciocalteu also indicates high phenolic concentrations in BJ and whole fruit extracts. These levels correlate with strong antioxidant activity measured through ORAC, DPPH, and ABTS assays across different processing methods, including spray-drying used to produce bergamot juice powders [14].

Bergamot leaves may also represent a valuable source of polyphenols compared with fruit-derived fractions. Targeted and semi-quantitative profiling indicates that leaf polyphenol fractions (BLPF) contain a qualitative profile comparable to fruit polyphenol fractions (BFPF), with potentially higher total phenolic content depending on the extraction method and analytical conditions [10]. In quantitative terms, the major flavonoid classes identified in fruit are present in leaves, often at higher concentrations when expressed within the BLPF. In particular, neoeriocitrin (~133.4 mg/g), naringin (~164.3 mg/g), neohesperidin (~145.0 mg/g), melitidin (~31.2 mg/g), and brutieridin (~59.8 mg/g) have been reported as major constituents of BLPF. This represents a notable increase relative to fruit extracts and suggests that leaves can be a valuable alternative source of these bioactive compounds for nutraceutical extraction [10].

### 2.2. Phenolic Acids and Other Polar Compounds

In addition to flavonoids, BBP contain a complex array of phenolic acids and polar hydrophilic metabolites that contribute to the overall functional properties of bergamot matrices. Major phenolic acid classes include hydroxycinnamic and hydroxybenzoic acids, which are mainly localized in the juice, albedo, and pulp, and are also present in the leaf matrix at lower concentrations [27,28]. In addition, bergamot contains non-phenolic organic acids, such as citric and malic acids, which contribute to juice acidity, sensory properties, and polyphenol stability.

Quantitative chromatographic profiling (HPLC-DAD and LC-MS/MS) has revealed that the dominant phenolic acids in BBP are caffeic acid, p-coumaric acid, ferulic acid, sinapic acid, and chlorogenic acid. These molecules occur either as free acids or as esters with quinic, or citric acid, forming stable conjugates that increase solubility and may contribute to antioxidant activity [29,30]. Average total phenolic acid concentration in BJ ranges from 60 to 120 mg gallic acid equivalents (GAE)/100 mL, while peel extracts can reach between 250 and 400 mg GAE/100 g dry weight (dw), depending on cultivar and extraction method [31].

In leaves, the same acids are present at 20–30% lower absolute concentrations but show higher specific antioxidant potency per mg of phenolic content [32].

Minor phenolic components identified by LC-ESI-MS/MS include vanillic, syringic, gallic, and protocatechuic acids, together accounting for less than 5% of total phenolics [28].

Besides phenolic acids, bergamot contains various polar constituents such as ascorbic acid, citric and malic acids, glycosylated flavones, and hydroxycinnamoyl derivatives of tyrosol and hydroxytyrosol [26].

These compounds enhance total reducing capacity and contribute to the fruit’s characteristic sour-bitter taste profile.

From a structural standpoint, phenolic acids in bergamot exhibit key electron-donating and resonance-stabilizing functional groups, notably the catechol moiety (–OH at C3′ and C4′) in caffeic acid and the methoxy substituents in ferulic and sinapic acids. These groups enhance redox potential and hydrogen atom transfer kinetics, making them efficient free-radical scavengers. The presence of the C=C–COOH conjugated system in hydroxycinnamates also allows chelation of transition metals such as Fe^3+^ and Cu^2+^, which is critical for inhibition of Fenton-type oxidative reactions in biological and food systems [29].

Bergamot’s polar matrix also includes abundant hydrophilic organic acids that influence both nutraceutical function and sensory quality. In BJ, ascorbic acid is present at concentrations approaching ~0.6 g/L, while citric acid is the dominant organic acid (36–49 g/L), playing an important role in juice acidity and polyphenol stability. The prominence of these organic acids defines the low pH typical of BJ and enhances its antioxidant potential through synergistic effects with polyphenols [31,33].

### 2.3. Limonoids, Coumarins, and Alkaloids

BBP, particularly seeds, peel, and pomace and non-volatile fractions from cold-pressed essential oil, are rich in structurally diverse secondary metabolites such as limonoids, coumarins/furanocoumarins, and alkaloids. Limonoids are highly oxygenated triterpenoid derivatives primarily localized in the seeds, peel, and albedo fractions of citrus fruits. The major limonoids identified in bergamot include limonin, nomilin, and their glucoside derivatives. Their concentration varies markedly according to the by-product fraction analyzed, cultivar, extraction procedure, analytical method, and chemical form considered. Seeds and peel generally represent the richest matrices, particularly in aglycone forms such as limonin and nomilin, whereas juice and pulp-derived fractions contain lower but still detectable levels, mainly as limonoid glucosides [25,34].

Quantitative studies indicate that limonoid-rich seed and peel extracts may contain substantial amounts of total limonoids, with limonin often representing the predominant compound within the aglycone fraction [34]. In contrast, glucosidic forms are more commonly reported in juice and pulp-derived by-products, highlighting the importance of clearly distinguishing between matrices and limonoid forms when comparing concentration data [25].

Mechanistically, limonoids have been shown to modulate Nrf2/HO-1 antioxidant defences while suppressing NF-κB- and MAPK-mediated inflammatory signalling, supporting their anti-inflammatory, chemopreventive, and metabolic protective potential [35].

Coumarins and furanocoumarins represent another significant class of heterocyclic phytoalexins in bergamot, particularly abundant in the non-volatile fraction of bergamot essential oil (BEO) and in fruit tissues. In bergamot, citropten (5,7-dimethoxycoumarin) and bergapten (5-methoxypsoralen) are among the principal linear furanocoumarins identified in peel extracts and cold-pressed essential oils, contributing to the characteristic bitterness and photochemical behaviour of these extracts. Furanocoumarins such as bergamottin (5-geranoxypsoralen) and related derivatives incorporate a furo [3,2-g]benzopyran scaffold and are well-recognized for their interaction with cytochrome P450 enzymes (CYPs), especially CYP3A4, which underlies clinically relevant food–drug interaction effects observed with grapefruit and bergamot consumption. These compounds also exhibit antimicrobial, anti-inflammatory, and anticancer properties, primarily through modulation of oxidative stress pathways and inhibition of cell proliferation [36].

Compared with other phytochemical classes, alkaloids in bergamot have been less extensively studied, but available phytochemical surveys indicate the presence of nitrogen-containing secondary metabolites consistent with indole and tryptamine derivatives in leaves and, to a lesser extent, in fruit tissues. LC–QTOF–MS analyses have detected synephrine, tryptamine, and indole derivatives in leaves and peel. Concentrations are typically 0.1–0.8 mg/g dw, with synephrine being predominant [37]. These compounds are biosynthesized from aromatic amino acids (e.g., tryptophan) and contribute to plant defence and stress response. Notably, BJ and peel extracts have been reported to contain quaternary ammonium compounds, which are polar alkaloid-like constituents that may modulate metabolic pathways and cellular signalling [38].

The relative abundance of limonoids, coumarins, and alkaloids in bergamot reflects tissue specificity and biosynthetic regulation. Limonoids and furanocoumarins are primarily sequestered in peel (flavedo and albedo) and seeds, where they contribute to the bitterness and deterring properties of the fruit. Their distribution in the juice fraction is lower but measurable when advanced LC-MS, HPLC-DAD, or GC-MS methods are applied to enriched extracts. In a comprehensive HPLC-DAD evaluation of BBP from three Calabrian cultivars, limonoid profiles exhibited distinct compound clusters consistent with known citrus limonoids, corroborating their high relative enrichment in non-juice tissues and validating the utility of fruit waste materials as a source of these bioactives [12].

### 2.4. Essential Oils and Volatile Compounds in Residual Matrices

Although BEO is primarily obtained from the flavedo during industrial processing, significant amounts of volatile compounds remain entrapped in peel residues and pomace. BEO is a chemically complex mixture, predominantly obtained by mechanical cold pressing, and is mainly composed of a volatile fraction, generally accounting for about 93–96% of the total oil, primarily including monoterpenes and oxygenated monoterpenoids. The remaining minor non-volatile fraction contains coumarins and furanocoumarins, such as bergapten and bergamottin [39].

The main volatile constituents identified through GC-MS and GC–FID analyses include limonene, linalyl acetate, and linalool, which together account for the majority of the volatile profile. Limonene typically represents 25–50% of the volatile fraction, while linalyl acetate ranges between 15 and 40% and linalool between 2 and 20% depending on cultivar, geographical origin, and extraction conditions [39].

However, during cold pressing or hydrodistillation, 85–99% of the oil is recovered from the flavedo, but peel and pomace still contain limonene, linalool, linalyl acetate and other mono/sesquiterpenes detected by GC/GC–MS in peels and rind extracts. Citrus processing residues are known to retain volatile terpenes due to matrix interactions and incomplete mass transfer during extraction.

These matrices are rich in structural polysaccharides such as pectin and cellulose, which can retain volatile terpenes through physical entrapment and adsorption phenomena. As a result, compounds such as limonene and linalool can persist in the solid residues even after primary extraction [40]. The coexistence of residual volatiles and phenolic compounds makes these matrices particularly relevant for valorisation strategies [41].

### 2.5. Correlation Between Phytochemical Composition and Biological Activity

The biological functionality of secondary metabolites of bergamot is deeply rooted in the complexity of its phytochemical composition. Each major class of bioactive molecules, flavonoids, phenolic acids, limonoids, coumarins, alkaloids, and volatile terpenoids, acts in a structurally coordinated manner, producing synergistic effects on redox homeostasis, lipid metabolism, inflammation, and detoxification. This multifaceted bioactivity stems from specific molecular interactions that can be attributed to the chemical architecture of these compounds, their substituent patterns, and their physicochemical properties [21]. Table 2 shows the correlation between major phytochemical classes of bergamot and their molecular and physiological activities (2023–2025).

A central determinant of this bioactivity is the close relationship between chemical structure and molecular function. Flavanone glycosides dominate the polyphenolic profile of bergamot and provide a substantial contribution to redox regulation through hydroxylation patterns, catechol moieties, and glycosylation, which together modulate solubility, reactivity, and interaction with biological targets. These same structural features explain both the high antioxidant potential of bergamot extracts and their technological challenges, including bitterness and limited bioavailability.

The presence of HMG-flavanones, namely brutieridin and melitidin, represents a distinctive biochemical trait of bergamot. Owing to their 3-hydroxy-3-methylglutaryl moiety, these compounds show statin-like structural features and have been associated with HMG-CoA reductase inhibition, providing a mechanistic basis for the lipid-lowering effects reported for bergamot preparations [42].

Importantly, the enrichment of these compounds in peel and albedo tissues highlights the strategic relevance of pomace as a source of cardiometabolically active ingredients.

Beyond flavonoids, phenolic acids and limonoids further shape the biological profile of bergamot matrices. Phenolic acids act synergistically with flavanones to enhance antioxidant efficacy and oxidative stability, particularly in complex food systems. Limonoids contribute additional redox-modulating and anti-inflammatory activity, but their strong bitterness and tissue-specific distribution introduce sensory and technological constraints that must be managed during extraction and formulation.

Coumarins and furanocoumarins add antimicrobial and enzyme-modulating properties but also raise safety considerations due to their interaction with cytochrome P450 enzymes. Together with minor constituents such as alkaloids and residual volatile terpenoids, these compounds contribute to the overall bioactive and sensory fingerprint of bergamot, reinforcing the need for controlled and selective recovery strategies.

Overall, the correlations between phytochemical composition and biological activity in bergamot support the view that preserving matrix complexity and synergistic interactions is often more advantageous than isolating single compounds. These considerations provide the mechanistic and conceptual framework for the green extraction technologies discussed in the following chapter, where process design is evaluated in light of phytochemical integrity, functionality, and food-grade applicability.

## 3. Green Extraction Technologies

The extraction of high value-added compounds from agri-food by-products, including bergamot (*Citrus bergamia*) by-products, is increasingly recognized as a strategic approach to enhance the recovery of target molecules while reducing waste streams and the overall environmental impact of processing chains [44]. The valorisation of plant-derived residues as sources of functional ingredients has therefore become a research priority [45].

Within this framework, green extraction technologies play a pivotal role. The integration of emerging extraction techniques with solvents selected for safety, renewability, and low environmental impact can substantially improve the balance between extraction efficiency and product quality [46].

The following sections critically compare the main green extraction options applied to BBP, with specific emphasis on food-grade applicability, techno-economic feasibility, extract safety, and their effectiveness in recovering carotenoids, polyphenols, and pectic polysaccharides.

### 3.1. Bergamot By-Products as Feedstocks for Green Extraction

The industrial processing of bergamot for essential oil and juice production generates significant amounts of solid residues, primarily referred to as pomace (pastazzo), which represent a valuable matrix for the recovery of bioactive compounds [12].

These by-products are mainly composed of peel (flavedo and albedo), residual pulp, and variable amounts of seeds, depending on processing conditions. From a technological perspective, the heterogeneous composition of bergamot residues plays a key role in defining extraction strategies. The peel includes an external pigmented layer (flavedo), rich in oil glands and partially depleted volatile compounds when derived from essential-oil extraction, and an inner albedo layer particularly rich in pectic polysaccharides. Both fractions also contain sugars, terpenes, and phenolic compounds [26,47,48]. Spent pulp is mainly composed of segment membranes and walls, whereas seeds are characterized by high levels of limonoids such as nomilin and limonin [26].

This compositional diversity requires the selection of extraction processes tailored to both the target compounds and the physicochemical characteristics of the matrix, in order to ensure selectivity, efficiency, and compatibility with food-grade applications. In this context, the recovery of BPF represents a relevant example of targeted valorisation, enabling the production of antioxidant-rich extracts suitable for functional food applications [49].

This matrix-driven approach is essential for designing efficient and scalable green extraction processes.

### 3.2. Conventional vs. Green Extraction Approaches

Conventional extraction techniques (CET), including solvent extraction and steam distillation, remain widely applied at the industrial level due to their robustness, scalability, and well-established process control. Acid or alkaline treatments may be used for specific purposes, such as pectin recovery or matrix disruption, but they are less suitable for food-grade polyphenol extraction because harsh pH conditions can promote flavonoid degradation. However, these approaches are often associated with high solvent consumption, elevated energy requirements, long processing times, and, in some cases, the use of non-food-grade or environmentally critical solvents. In addition, conventional processes may show limited selectivity toward specific classes of compounds, particularly when applied to complex matrices such as BBP, where polyphenols, pectins, and lipophilic constituents coexist within structurally heterogeneous tissues. As a result, extraction efficiency and product quality are strongly influenced by process parameters, including solvent type, temperature, solid-to-liquid ratio, and extraction time [50,51].

To address these limitations, increasing attention has been directed toward green extraction strategies, which aim to reduce environmental impact while improving process efficiency and product functionality. In this context, green extraction refers not only to the replacement of conventional solvents with safer and more sustainable alternatives (e.g., water, ethanol, or deep eutectic solvents), but also to the application of process intensification technologies capable of enhancing mass transfer and reducing extraction time and energy consumption [52].

Among these approaches, ultrasound-assisted extraction (UAE), microwave-assisted extraction (MAE), enzyme-assisted extraction (EAE), and supercritical fluid extraction (SFE) have been widely investigated for the recovery of bioactive compounds from plant matrices, including citrus by-products. These technologies can improve extraction selectivity and yield while enabling milder processing conditions, which are particularly relevant for preserving thermolabile compounds.

Nevertheless, the selection of extraction technology must be tailored to the specific characteristics of the matrix and target compounds, while ensuring compatibility with food-grade requirements and process scalability.

### 3.3. Ultrasound-Assisted Extraction (UAE)

UAE is a non-conventional technology often framed as a “green” option because it can enhance process efficiency by reducing solvent use, processing time and energy demand while maintaining or even improving extraction yields [53]. Its effectiveness is mainly attributed to acoustic cavitation, which promotes cell disruption and facilitates the release of intracellular compounds into the extraction medium [54].

Compared with CET, UAE enables efficient recovery of thermolabile compounds under relatively mild conditions, limiting thermal degradation and improving the preservation of bioactive molecules such as polyphenols and flavonoids [55,56]. However, extraction performance is strongly dependent on process parameters, including acoustic intensity, solvent composition, solid-to-liquid ratio, and temperature, which require careful optimization depending on the target compounds and matrix characteristics [57].

In the case of BBP, UAE has been identified as a particularly suitable strategy for the recovery of polyphenol-rich fractions using food-grade solvents such as water and aqueous ethanol. Experimental studies on bergamot pomace have demonstrated that UAE can achieve high extraction yields and antioxidant activity under milder conditions compared with CET [12]. This aspect is especially relevant for preserving the functional properties of bergamot-derived bioactives intended for food applications.

Nevertheless, some limitations should be considered. Excessive ultrasound intensity or prolonged treatment times may lead to degradation of sensitive compounds or the co-extraction of undesired components, potentially affecting extract quality and sensory properties. In addition, scale-up remains a critical challenge, as energy distribution and cavitation effects are more difficult to control in industrial systems.

Comparable improvements in phenolic recovery and process efficiency have been reported for other citrus by-products, supporting the broader applicability of UAE in citrus waste valorisation [58].

However, the effectiveness of this approach remains highly matrix-dependent, reinforcing the need for process optimization tailored to specific raw materials and target compounds.

### 3.4. Microwave-Assisted Extraction (MAE)

MAE is a process intensification technique that enhances the release of bioactive compounds from plant matrices through rapid volumetric heating, particularly effective in polar and moderately polar systems [59,60].

Compared with CET, MAE significantly reduces extraction time and solvent consumption while often achieving comparable or higher yields.

The improved performance of MAE is associated with rapid heating of both the solvent and the hydrated matrix, which promotes cell disruption, increased porosity, and enhanced mass transfer. These features make MAE particularly suitable for the recovery of compounds such as polyphenols and pectic polysaccharides from complex plant matrices.

However, the efficiency of MAE is highly dependent on process parameters, including solvent dielectric properties, irradiation power, temperature, and extraction time. In particular, excessive temperatures or prolonged exposure may lead to degradation of thermolabile compounds and modification of sensitive bioactive structures, representing a critical limitation for food applications.

In citrus waste valorisation, MAE is commonly performed using water or hydroalcoholic mixtures to ensure food-grade compatibility. It can also be integrated into cascade processing schemes, enabling multi-product recovery within shorter processing times compared with conventional approaches [61].

Studies on citrus residues, including orange and lemon peels, have demonstrated improved flavonoid recovery and enhanced pectin extraction efficiency compared with conventional heating methods [62].

In the case of BBP, MAE has been explored both as a standalone technique and in comparison, with other green extraction methods. Experimental studies on BBP using short treatment times (5–15 min) and moderate microwave power (250–800 W) have demonstrated rapid recovery of characteristic flavonoids, including brutieridin and melitidin, confirming the suitability of MAE for the valorisation of bergamot-derived bioactive compounds [12].

Compared with UAE, MAE may present lower process flexibility and a higher risk of localized overheating, particularly in heterogeneous matrices. In addition, scale-up remains challenging due to non-uniform energy distribution and difficulties in controlling temperature gradients at industrial scale.

### 3.5. Enzyme-Assisted Extraction (EAE)

EAE is based on the use of cell wall-degrading enzymes, such as pectinases, cellulases, and hemicellulases, to selectively hydrolyse structural polysaccharides and enhance the release of intracellular compounds under mild, typically aqueous conditions [61]. By increasing tissue permeability and reducing structural barriers, enzymatic treatments facilitate mass transfer and improve extraction efficiency.

This approach is particularly relevant for citrus by-products, where the high content of pectic polysaccharides can limit solvent penetration and complicate downstream operations such as filtration and clarification. Controlled enzymatic depolymerization improves both the accessibility of target compounds and the rheological properties of the extraction medium, supporting more efficient solid–liquid separation.

In addition to improving polyphenol recovery, enzymatic pretreatments have been shown to enhance essential oil extraction by promoting oil gland disruption and reducing matrix viscosity [63].

In BBP, EAE has been applied within integrated processing schemes aimed at recovering polyphenol-rich fractions and improving subsequent stabilization and formulation steps [21].

However, several limitations should be considered. Enzymatic processes typically require longer treatment times compared with physical intensification techniques such as UAE or MAE, and their efficiency is strongly influenced by process conditions including pH, temperature, and enzyme dosage. In addition, enzyme cost, potential variability in enzyme activity, and the need for process control may limit large-scale industrial implementation.

Compared with purely physical extraction techniques, EAE offers higher selectivity and milder processing conditions, but often at the expense of longer processing times and increased operational complexity. For this reason, EAE is frequently employed as a pretreatment step in combination with other extraction technologies, rather than as a standalone process.

### 3.6. Supercritical Fluid Extraction (SFE)

SFE, most commonly employing supercritical CO_2_, is widely regarded as a green-leaning separation technology because it limits the use of conventional organic solvents while relying on an inert and recyclable processing fluid, with rapid solvent removal upon depressurization [59].

The solvating power of supercritical CO_2_ can be finely tuned by adjusting pressure and temperature, allowing selective extraction of target compounds through density modulation. This makes SFE particularly suitable for recovering non-polar and moderately polar compounds, such as volatile fractions, lipids, and waxes, under relatively mild thermal conditions compatible with heat-sensitive molecules [59,64].

However, the extraction of more polar compounds, such as polyphenols, typically requires the addition of co-solvents (e.g., ethanol), which increases process complexity and introduces additional considerations related to solvent recovery and regulatory compliance in food applications [50,59].

In citrus matrices, SFE is frequently used as a selective front-end step for recovering lipophilic fractions, potentially followed by aqueous or hydroalcoholic extraction of phenolics and pectins within integrated biorefinery schemes [59,61].

In the case of bergamot, SFE has been primarily investigated for the selective extraction and fractionation of essential oil components from peel, enabling high-purity recovery of volatile compounds while preserving their sensory and functional properties.

Compared with other green extraction methods such as UAE or MAE, SFE offers higher selectivity and solvent-free extracts for lipophilic compounds but requires more complex process conditions and infrastructure. For this reason, it is most effectively applied within integrated extraction schemes, where it serves as a first step in the fractionation of complex matrices.

### 3.7. Natural Deep Eutectic Solvents (NADES)

In recent years, deep eutectic solvents (DES), and particularly NADES, have gained increasing attention as alternative extraction media to conventional organic solvents [65].

These systems are typically formed by combining natural components acting as hydrogen-bond donors and acceptors, resulting in eutectic mixtures with tuneable polarity, low volatility, and the potential to be designed for specific extraction targets [65,66].

From an extraction perspective, NADES offer high solubilization capacity for a wide range of bioactive compounds, particularly polyphenols, and can be tailored to improve selectivity depending on their composition. This flexibility makes them attractive for the recovery of compounds from complex matrices such as citrus by-products.

However, the classification of NADES as “green” solvents should be considered with caution. Although their individual components are often of natural origin, the safety of the final mixtures is not automatically guaranteed. Toxicological evaluation, residual solvent assessment, and regulatory compliance remain critical requirements before their application in food systems [63,67].

A major operational limitation of NADES is their high viscosity, which can significantly restrict mass transfer and reduce extraction efficiency in solid matrices. To overcome this limitation, process optimization strategies such as controlled water addition, temperature adjustment, or coupling with intensification techniques (e.g., UAE or MAE) are commonly applied [68,69].

In citrus by-products, NADES-based extraction has demonstrated improved recovery of phenolic compounds compared with conventional solvents, although performance is strongly dependent on solvent composition and process conditions [70].

In the case of BBP, their application remains relatively limited but represents a promising area for future research, particularly for the selective extraction of polyphenol-rich fractions under mild conditions.

Compared with conventional organic solvents, NADES offer advantages in terms of tunability and reduced volatility, but their practical implementation in food applications is still constrained by viscosity, process optimization requirements, and the need for comprehensive safety assessment.

### 3.8. Integration, Optimization and Industrial Applicability

The recovery of bioactive compounds from agri-food by-products is increasingly moving toward integrated extraction strategies, in which multiple techniques are combined to overcome the limitations of single-step processes. This approach is particularly relevant for complex matrices such as bergamot residues, where lipophilic compounds, polyphenols, and pectic polysaccharides coexist and require different extraction conditions.

In this context, sequential or hybrid processing schemes enable the selective fractionation of target compounds under controlled conditions. For example, lipophilic fractions can be first recovered using non-polar extraction techniques, followed by the extraction of polar compounds through aqueous or hydroalcoholic systems.

Hybrid configurations, including the coupling of NADES with ultrasound or microwave-assisted extraction, as well as the integration of enzymatic pretreatments, membrane filtration, or adsorption-based purification, may further improve process efficiency while reducing solvent consumption and downstream processing requirements [71].

From a critical perspective, the available literature indicates that no single green extraction technology can be considered universally optimal for BBP. UAE and MAE are attractive for phenolic compound recovery because they reduce extraction time and solvent use, but their scale-up requires careful control of energy distribution, temperature, and process reproducibility. EAE provides higher selectivity under mild conditions, particularly for pectin-rich matrices, although enzyme cost, longer processing times, and strict control of operational parameters may limit its industrial use. SFE is suitable for lipophilic and volatile fractions and can produce solvent-free extracts, but high capital costs and lower efficiency for polar compounds restrict its application to high-value products or integrated biorefinery schemes.

NADES-based extraction is promising for the recovery of polyphenols and other polar compounds; however, its application in food systems is still limited by high viscosity, solvent removal or retention, toxicological assessment, and regulatory acceptance. Therefore, despite their potential environmental advantages, further safety validation is required before NADES can be routinely used in food-grade formulations.

Overall, the practical implementation of green extraction technologies for BBP should not be assessed only in terms of extraction yield. Future studies should also consider techno-economic feasibility, process scalability, life-cycle impact, sensory consequences, safety assessment, and regulatory compliance in real food systems. These aspects are essential to determine whether laboratory-scale processes can be translated into industrial applications.

A comparative overview of the main green extraction technologies applied to BBP, including their solvent systems, target compounds, advantages, limitations, and food-grade suitability, is presented in Table 3.

From a critical perspective, the available literature indicates that no single green extraction technology can be considered universally optimal for the valorisation of BBP. Each approach presents specific advantages and limitations depending on the target compounds, matrix composition, intended food application, and level of technological maturity. UAE and MAE are particularly attractive for the recovery of phenolic compounds due to their relatively short extraction times, reduced solvent requirements, and compatibility with aqueous or hydroalcoholic food-grade solvents. However, their industrial implementation requires careful control of energy distribution, temperature, and process reproducibility, especially when heterogeneous matrices such as bergamot pomace are used.

EAE offers high selectivity and mild operating conditions, making it suitable for matrices rich in pectic polysaccharides, but its practical application may be limited by enzyme cost, longer processing times, and the need for strict control of pH, temperature, and enzyme activity. SFE provides high-quality solvent-free extracts and is particularly appropriate for lipophilic and volatile fractions, although its high capital cost, operational complexity, and lower efficiency for polar compounds may restrict its use to high-value applications or integrated biorefinery schemes.

NADES-based extraction represents a promising strategy for improving the recovery of polyphenols and other polar compounds. Nevertheless, its translation into food applications remains less advanced because of issues related to viscosity, solvent removal or retention, toxicological assessment, and regulatory acceptance. Therefore, despite their environmental potential, NADES require further safety validation before routine use in food-grade formulations.

Overall, the most realistic industrial scenario appears to be the development of integrated or sequential extraction processes, in which different technologies are combined according to the chemical nature of the target fractions. Such an approach may improve extraction selectivity, reduce waste generation, and increase the economic viability of BBP valorisation. Future studies should therefore move beyond laboratory-scale extraction yield and include techno-economic analysis, life-cycle assessment, process scalability, sensory impact, safety evaluation, and regulatory feasibility in real food systems.

## 4. Stabilization and Formulation of Bergamot Extracts

The increasing interest in sustainable functional ingredients has stimulated the exploration of agri-food by-products as sources of bioactive compounds. Among citrus residues, BBP, particularly pomace, represent a promising matrix for the recovery of compounds with technological and nutraceutical value.

However, the effective exploitation of bergamot-derived bioactives requires appropriate stabilization and formulation strategies to maintain their functional integrity during processing, storage, and incorporation into food systems. Polyphenols, flavonoids, limonoids, and volatile constituents present in bergamot extracts are particularly sensitive to environmental conditions such as oxygen exposure, light, temperature fluctuations, pH variations, and interactions with food matrix components [29]. These factors may lead to degradation, loss of antioxidant capacity, and deterioration of sensory properties, ultimately limiting their practical application in functional foods.

For this reason, the development of suitable delivery and stabilization systems has become a key research area, aiming to enhance the stability, bioavailability, and controlled release of bergamot bioactive compounds (BBC). An overview of these approaches is presented in Table 4.

### 4.1. Microencapsulation Techniques

Microencapsulation represents a crucial technology for protecting bergamot bioactives from environmental degradation while facilitating their controlled release and integration into diverse food matrices. This process involves the entrapment of active ingredients within protective coating materials, resulting in particles typically ranging from 1 to 1000 μm in diameter [72].

#### 4.1.1. Spray-Drying

Spray-drying was the most widely adopted microencapsulation technique for bergamot extracts due to its cost-effectiveness, industrial scalability, and compatibility with food-grade materials [73]. The process involves atomizing a feed emulsion containing bergamot bioactive and wall materials into a heated chamber, where rapid solvent evaporation generates dry microparticles. Recent studies have demonstrated the effectiveness of spray-drying for stabilizing BPF and vitamin C [14].

The selection of appropriate encapsulating agents plays a crucial role in determining encapsulation efficiency and stability. Maltodextrin is frequently used because of its neutral taste, low viscosity, good film-forming properties, and helps to preserve the polyphenol content and antioxidant activity of bergamot [74].

Gum arabic is widely recognized for its excellent emulsifying properties derived from its protein-polysaccharide structure and has demonstrated effective retention of volatile compounds and oxidative protection [75]. Similarly, octenyl succinic anhydride (OSA)-modified starches provide improved emulsifying ability and encapsulation performance compared to native starches [76]. Recent investigations on bergamot extract microencapsulation have reported encapsulation efficiencies ranging from 75% to 95% when using optimized maltodextrin–gum arabic combinations with inlet temperatures of 150–180 °C and outlet temperatures of 70–90 °C [77]. However, high inlet temperatures may promote the degradation of thermolabile polyphenols and volatile compounds; therefore, process conditions should be optimized to balance encapsulation efficiency and bioactive retention.

#### 4.1.2. Freeze-Drying

Freeze-drying is particularly suitable for thermolabile compounds that may undergo degradation during high-temperature processes such as spray-drying [73].

In this process, frozen extracts are dehydrated through sublimation under reduced pressure, allowing preservation of sensitive phytochemicals.

Common carriers used in freeze-dried bergamot formulations include maltodextrin, trehalose, inulin, and protein–polysaccharide systems [78]. Among these, trehalose has shown improved stability of citrus polyphenols during storage due to its protective effect against oxidative and structural degradation [78]. Moreover, the porous structure of freeze-dried powders promotes rapid dissolution and efficient release of encapsulated compounds, making them suitable for instant beverage formulations and nutraceutical applications [79].

#### 4.1.3. Complex Coacervation

Complex coacervation is based on electrostatic interactions between oppositely charged biopolymers, typically proteins and anionic polysaccharides, resulting in the formation of a polymer-rich phase capable of encapsulating hydrophobic bioactives [80].

For BEO, gelatin–gum arabic systems have demonstrated excellent encapsulation performance due to the formation of stable coacervate shells around oil droplets [81].

Microcapsules produced through this technique exhibit high encapsulation efficiencies and improved resistance to oxidation and volatilization compared with conventional spray-dried systems [81].

#### 4.1.4. Emerging Encapsulation Technologies

Recent technological advances have introduced novel encapsulation strategies capable of producing nano- and micro-structured delivery systems with improved control over morphology and release behaviour. Electrospinning and electrospraying techniques exploit high-voltage electric fields to generate nanofibers or nanoparticles from polymer solutions [82].

Food-grade polymers such as zein, pullulan, and cellulose derivatives have been successfully used to fabricate electrospun nanofibers incorporating bergamot bioactives, showing enhanced stability and controlled release profiles [83]. In particular, coaxial electrospinning allows the production of core–shell nanostructures that provide additional protection for sensitive compounds [84].

Supercritical anti-solvent (SAS) precipitation and particles from gas-saturated solutions (PGSS) represent supercritical fluid-based encapsulation alternatives that operate under mild temperature conditions while avoiding organic solvent residues. These techniques have been successfully applied to citrus bioactive encapsulation, producing fine particles with narrow size distributions and high bioactivity retention [85].

### 4.2. Nanoemulsions and Lipid-Based Delivery Systems

Nanoemulsions, characterized by droplet sizes typically below 200 nm, represent kinetically stable colloidal dispersions that offer significant advantages for delivering lipophilic bergamot constituents. The extremely small droplet size increases optical transparency, enhances physical stability against gravitational separation, and improves bioavailability through increased interfacial area and enhanced cellular uptake [86].

#### 4.2.1. Nanoemulsions

Nanoemulsions can be produced using high-energy techniques such as high-pressure homogenization, microfluidization, and ultrasonication, or through low-energy methods based on physicochemical phenomena such as phase inversion temperature (PIT) or spontaneous emulsification [87].

For BEO, recent studies have demonstrated successful nanoemulsion formation using food-grade emulsifiers including modified starches, whey protein isolate, lecithin, and Tween 80. Optimized formulations typically employ oil phase concentrations of 5–20% and surfactant-to-oil ratios of 0.1–0.5 to achieve stable nanoemulsions with acceptable sensory properties [88].

Studies have highlighted potential applications in non-food sectors, too. In fact, bergamot peel from food processing waste has proved useful in the design of lipid-based nanosystems intended for topical application [89].

#### 4.2.2. Solid Lipid Nanoparticles and Nanostructured Lipid Carriers

Solid lipid nanoparticles (SLN) and nanostructured lipid carriers (NLC) represent alternative lipid-based delivery platforms particularly suited for lipophilic bergamot bioactives [90].

Recent applications to citrus bioactives have shown that SLN/NLC systems enhance oxidative stability, provide controlled release, and improve oral bioavailability through lymphatic absorption pathways [91]. For BEO and limonoids, NLC formulations have demonstrated improved encapsulation efficiency and sustained release profiles compared to SLN [89]. Pawar et al. [92] demonstrated that the incorporation of bergamottin into SLNs constitutes a safe and efficient delivery system, suggesting potential applications both in pharmacology and in the food field.

#### 4.2.3. Liposomal Delivery Systems

Liposomes, composed of phospholipid bilayers forming vesicular structures, represent versatile carriers capable of encapsulating both hydrophilic and lipophilic bergamot constituents [93]. Several liposomal formulations have been investigated for citrus bioactive delivery, including multilamellar vesicles (MLV), small unilamellar vesicles (SUV), and large unilamellar vesicles (LUV) [94]. Liposomal bergamot extracts have shown promise for functional beverage applications, providing optical clarity, enhanced stability, and improved sensory characteristics compared to conventional emulsions [11].

### 4.3. Biopolymer and Protein-Based Carriers

Biopolymers have attracted increasing interest as food-grade carriers for the stabilization and delivery of bergamot-derived bioactive compounds. Their natural origin, biodegradability, and compatibility with food systems make them particularly suitable for functional food and nutraceutical applications. Among them, polysaccharides and proteins are widely employed due to their ability to form films, hydrogels, nanoparticles, and emulsifying structures capable of entrapping phytochemicals and improving their stability during processing and storage [95]. These macromolecules can also interact with bioactive compounds through electrostatic interactions, hydrogen bonding, and hydrophobic forces, enabling the design of delivery systems with tailored release behaviour depending on the food matrix and gastrointestinal conditions.

#### 4.3.1. Polysaccharide-Based Encapsulation Systems

Polysaccharides are widely used as encapsulating materials because of their excellent film-forming ability, viscosity-modifying properties, and capacity to interact with bioactive compounds through hydrogen bonding and electrostatic interactions.

Alginate is among the most employed polysaccharides for encapsulation applications. This anionic polymer forms hydrogels through ionic cross-linking with divalent cations such as calcium ions, generating stable matrices capable of entrapping polyphenols and essential oil droplets. Alginate beads have been successfully used to encapsulate citrus polyphenols, providing pH-responsive release behaviour suitable for targeted intestinal delivery [96].

Chitosan represents another widely studied polysaccharide derived from chitin. It exhibits antimicrobial activity, mucoadhesive properties, and pH-responsive behaviour, making it particularly suitable for food and nutraceutical applications. Chitosan-based nanoparticles and films have demonstrated promising results for the stabilization and controlled release of citrus-derived bioactive compounds [97].

Cyclodextrins, cyclic oligosaccharides with hydrophobic cavities, form inclusion complexes with lipophilic bergamot constituents, enhancing their aqueous solubility and stability. Beta-cyclodextrin and its derivatives complex with BEO monoterpenes and flavonoid aglycones, protecting them from oxidation and volatilization while improving water dispersibility [98].

Cellulose derivatives, including methylcellulose, hydroxypropyl methylcellulose, and carboxymethylcellulose, provide film-forming and viscosity-modifying properties valuable for bergamot extract stabilization [99].

#### 4.3.2. Protein-Based Delivery Systems

Proteins offer complementary advantages to polysaccharides as encapsulation materials, including superior emulsifying capacity, gelation properties, and enhanced nutritional value. The amphiphilic nature of proteins, arising from the presence of both hydrophilic and hydrophobic amino acid residues, enables effective interfacial stabilization of oil-in-water emulsions [100]. Whey proteins represent one of the most extensively studied protein carriers. Their ability to undergo thermal denaturation and aggregation enables the formation of three-dimensional networks capable of entrapping bioactive compounds. Whey protein nanoparticles have demonstrated effective encapsulation of citrus polyphenols, improving their stability during processing and storage while potentially enhancing gastrointestinal bioavailability [12].

Caseins and caseinates are also widely employed due to their strong emulsifying properties and flexible molecular structure. Sodium caseinate can stabilize emulsions containing citrus essential oils and serves as an efficient carrier during subsequent encapsulation processes such as spray-drying. The open conformation of casein molecules facilitates interactions with polyphenols through hydrophobic interactions and hydrogen bonding [13].

Gelatin exhibits unique thermogelation properties and is particularly valued in complex coacervation systems. The isoelectric point of gelatin (pH 4.7–5.2) enables coacervation with anionic polysaccharides under acidic conditions, forming robust capsule walls around BEO droplets [14].

In recent years, plant-derived proteins have gained increasing attention as sustainable alternatives to animal-based proteins. Among these, zein, a hydrophobic maize protein, exhibits excellent film-forming properties and the ability to self-assemble into nanoparticles. Zein-based nanostructures have been successfully employed for encapsulating citrus bioactive compounds, improving their stability and enabling controlled release in food and nutraceutical applications [6].

### 4.4. Stability and Bioavailability Considerations

The chemical stability of BBC plays a crucial role in determining their shelf life and functional performance when incorporated into food products. Polyphenol degradation generally follows first-order or pseudo-first-order kinetics, with reaction rates influenced by environmental parameters such as temperature, pH, oxygen availability, water activity, and the presence of pro-oxidant or antioxidant compounds. Similarly, the retention of volatile constituents from BEO in encapsulated systems depends on diffusion processes through the wall matrix, partition coefficients between oil and carrier phases, and the structural integrity of the encapsulation system [9].

In encapsulated systems, surface oil content is considered a critical parameter affecting oxidative stability and volatile retention. Surface oil represents the fraction of essential oil not fully entrapped within the encapsulating matrix and is therefore more susceptible to oxidation and evaporation. For optimal stability, surface oil levels are generally maintained below 5% through appropriate process optimization and selection of encapsulating materials [16].

Food processing conditions can also significantly affect the stability of bergamot bioactives. Thermal treatments, such as baking processes typically performed at 150–200 °C for 10–30 min, may lead to degradation of polyphenols and volatile compounds. However, spray-dried powders containing suitable wall materials often exhibit improved thermal resistance, as the encapsulation matrix provides partial protection against direct heat exposure while the low moisture content limits degradation reactions [17].

More severe technological processes, such as extrusion, represent additional challenges due to the combined effects of elevated temperature (100–180 °C), mechanical shear, and pressure. In these conditions, pre-encapsulation strategies have been shown to substantially enhance bioactive retention. Studies report survival rates of approximately 60–85% for encapsulated polyphenols compared with 20–40% for non-encapsulated forms. The selection of heat-resistant carrier materials and careful optimization of extrusion parameters, including temperature, moisture content, and screw speed, are therefore essential for preserving the functional properties of bergamot extracts [18].

Once incorporated into foods, interactions between bergamot bioactives and food matrix components can further influence both stability and functional activity. In acidic beverages (pH 2.5–4.0), polyphenols may undergo acid-catalyzed hydrolysis of glycosidic bonds, generating aglycone forms that exhibit different stability and bioavailability profiles. Encapsulation systems can partially mitigate this degradation by providing a buffering microenvironment, although the effectiveness of this protection depends on the physicochemical properties of the wall materials, including their pH-dependent solubility and swelling behaviour [19].

Interactions with food proteins can both stabilize and destabilize bergamot polyphenols. While protein binding may reduce oxidative degradation, excessive interaction can cause aggregation and precipitation, particularly at pH values near protein isoelectric points [20]. In this context, the formation of soluble protein–polyphenol complexes may represent an optimal condition, improve compound stability while maintaining dispersion within the food matrix. In lipid-containing foods, oxidation processes may further influence the stability of bergamot antioxidants. Lipid oxidation generates reactive carbonyl species and free radicals capable of degrading polyphenolic compounds, leading to competitive oxidation phenomena. Additionally, the presence of transition metal ions can accelerate both lipid and polyphenol oxidation, highlighting the importance of chelation strategies or the use of encapsulating materials with metal-binding properties [21,22].

Beyond stability considerations, the bioavailability of bergamot lipophilic compounds, including essential oil components and flavonoid aglycones, is strongly influenced by digestive processes. These compounds require solubilization in mixed micelles formed during lipid digestion to become available for intestinal absorption. Consequently, the co-ingestion of dietary lipids or the use of lipid-based delivery systems can significantly enhance their bioaccessibility by promoting micelle formation during gastrointestinal digestion [23].

**Table 4:** Delivery systems for bergamot bioactive.

Category	Delivery System	Main Function	Main Materials	References
Microencapsulation	Spray-drying	Dry powders and protection during storage	Maltodextrin, gum arabic, modified starches, proteins	[72,73,75,76,77,101,102]
Microencapsulation	Freeze-drying	Protection of thermolabile compounds	Maltodextrin, trehalose, inulin, protein-polysaccharide combinations	[73,78,79]
Microencapsulation	Complex Coacervation	Encapsulation of hydrophobic bioactives	Gelatin + gum arabic	[80,81]
Microencapsulation	Electrospinning/Electrospraying	Nanofibers or nanoparticles with controlled release	Zein, pullulan, cellulose derivatives	[82,83,84]
Microencapsulation	Supercritical Anti-Solvent processes	Particle formation under mild conditions	Lipid or polymer matrices	[85,86]
Lipid-Based Systems	Nanoemulsions	Improved dispersion and bioaccessibility of lipophilic compounds	Modified starches, whey protein isolate, lecithin, Tween80	[87,89]
Lipid-Based Systems	Solid Lipid Nanoparticles	Protection and controlled release of lipophilic compounds	Triglycerides, fatty acids, waxes	[90,91]
Lipid-Based Systems	Nanostructured Lipid Carriers	Improved loading capacity	Solid + liquid lipids	[90,91]
Lipid-Based Systems	Liposomes	Encapsulation of hydrophilic and lipophilic compounds	Phospholipids	[93,94,101,103]
Polysaccharide-Based Systems	Alginate Beads	pH-responsive release	Calcium alginate	[96]
Polysaccharide-Based Systems	Chitosan Systems	Mucoadhesive and antimicrobial films or particles	Chitosan	[97]
Polysaccharide-Based Systems	Cyclodextrin complexes	Improved solubility and protection of volatile/lipophilic compounds	Beta-cyclodextrin and derivatives	[98]
Polysaccharide-Based Systems	Cellulose Derivatives	Film formation and viscosity modulation	MC, HPMC, CMC	[99]
Protein-Based Systems	Whey Proteins	Encapsulation and nanoparticle formation	Whey protein	[100,104]
Protein-Based Systems	Caseins/Caseinates	Emulsification and spray-drying support	Sodium caseinate	[105]
Protein-Based Systems	Gelatin	Thermogelling protein used in coacervation systems	Gelatin	[105,106]
Protein-Based Systems	Zein	Nanoparticles and nanofibers for controlled release	Zein	[107]
Hybrid and Co-Encapsulation Systems	Protein + Polysaccharide Systems	Combined emulsifying and barrier effects	Whey + maltodextrin; caseinate + gum arabic/modified starches	[100,108,109]
Hybrid and Co-Encapsulation Systems	Multilayer Emulsions	Co-encapsulation of polyphenols and essential oils	Multilayer emulsion systems	[110]
Hybrid and Co-Encapsulation Systems	Pickering Emulsions	Particle-stabilized emulsions	Cellulose nanocrystals	[102,111]

## 5. Food Applications of Bergamot-Derived Compounds

In recent years, bergamot and its processing by-products have attracted increasing interest as potential ingredients for food applications because of their complex phytochemical composition and possible technological value. These matrices contain polyphenols, flavonoids, dietary fibres, pectins, volatile compounds, and residual essential oil constituents, which may contribute to antioxidant protection, microbial control, flavour modulation, texture improvement, and functional enrichment. However, their implementation into real food matrices remains only partially explored. Most available studies have focused on chemical composition, biological activity, or nutraceutical potential, whereas fewer investigations have evaluated their behaviour during formulation, processing, and storage. This represents a relevant limitation, since the effectiveness of bergamot-derived ingredients depends not only on their bioactive profile, but also on their interaction with food components and their impact on product quality.

From a technological perspective, polyphenol-rich extracts may support oxidative stability and shelf-life extension, while fibre- and pectin-rich fractions can influence water retention, viscosity, and structure. At the same time, volatile compounds and residual essential oil fractions may provide characteristic citrus notes, but excessive bitterness, acidity, astringency, or intense aroma may negatively affect consumer acceptability. Therefore, dosage optimization, stabilization approaches, and appropriate formulation strategies are essential to balance functionality and sensory compatibility.

Overall, the use of BBP in food applications should be assessed through an integrated approach that considers technological performance, shelf-life effects, sensory acceptance, safety aspects, and regulatory constraints. These factors are crucial to determine whether bergamot-derived ingredients can move from promising bioactive sources to reliable components for industrial food production.

### 5.1. Bergamot Extracts as Natural Antioxidants

Several studies have investigated the antioxidant activity of bergamot extracts, evaluating both purified bioactive molecules, such as neohesperidin, naringin, neoeriocitrin, melitidin, brutieridin, and peripolin [112], and complex phenolic extracts obtained from bergamot tissues and by-products [10,23]. The strong radical-scavenging activity of bergamot flavonoids has been widely demonstrated through in vitro assays including ABTS, DPPH, and FRAP. Siano et al. [23] reported particularly high antioxidant capacity in BBP, which was closely correlated with their flavonoid content. Their study evaluated the antioxidant potential of several bergamot fractions, including edible tissues, first-pressing juice, and second-pressing juice, highlighting the high phenolic richness of these matrices and their potential for valorisation as functional food ingredients. The use of bergamot-derived antioxidants in food systems has been explored particularly in lipid matrices, where the prevention of lipid oxidation represents a major technological challenge. In this context the replacement of synthetic antioxidants with natural alternatives is increasingly demanded by consumers and industry.

Gattuso et al. [20] demonstrated that the incorporation of bergamot phenolic extracts into vegetable fat significantly increased total phenolic content, flavonoid concentration, and antioxidant activity. The enriched fat was subsequently used in biscuit formulations, resulting in baked products with improved oxidative stability compared to control samples. Importantly, the phenolic compounds retained their antioxidant properties even after the baking process, indicating their stability under thermal processing conditions.

Microencapsulated bergamot pomace extracts have also been applied as natural antioxidants in lipophilic food matrices [74]. When incorporated into sunflower oil, the microencapsulated extract significantly enhanced antioxidant capacity and improved oxidative stability during storage at elevated temperature (25 °C). The controlled release of phenolic compounds from the encapsulation matrix contributed to prolonged protection against lipid oxidation, supporting their application for shelf-life extension.

Another interesting application involves the enrichment of extra virgin olive oil with bergamot-derived compounds. Custureri et al. [113] investigated the incorporation of bergamot during olive oil processing, either through the addition of fresh bergamot during olive crushing or by infusion of freeze-dried bergamot. In both cases, the phenolic fraction enhanced antioxidant activity, reduced lipid oxidation, and better preservation of colour and quality during storage. Notably, the addition of bergamot during olive crushing resulted in stronger inhibition of lipid oxidation, confirming the effectiveness of bergamot polyphenols in lipid-rich matrices.

### 5.2. Use as Flavouring and Aromatizing Agent

Compared with other citrus fruits, the recovery of flavouring compounds from bergamot pomace has received relatively limited attention. After essential oil extraction, bergamot pomace is mainly used for pectin recovery, polyphenol extraction, or animal feed, and few studies have explored its potential for further recovery of aroma compounds for food flavouring purposes. Nevertheless, the aromatic profile of bergamot, characterized by intense citrus and floral notes, suggests relevant potential as a natural flavouring agent. Multari et al. [114] identified high concentrations of volatile compounds such as limonene, γ-terpinene, and α-pinene in citrus pomace, which contribute significantly to citrus-like aroma profiles.

Commercial beverages labelled as bergamot flavoured products are currently available. This is supported by an investigation conducted on commercial beers “flavoured with bergamot.” Although the typical citrus-floral flavour of bergamot is sought, the insights reported by Trovato et al. [115] showed that the aromatic profiles of the beers are not compatible with those of the essential oil, nor with the juice. The absence of linalyl acetate in beers led the authors to consider the use of artificial or alternative citrus aroma sources.

Two groups of researchers have focused on the evaluation of the aromatic effect of bergamot in extra virgin olive oil. A volatile profile validation study using chemical authentication was conducted on a commercial bergamot-flavoured oil [116]. The researchers, considering the concentrations of (+)-linalool, (+)-linalyl acetate, suggested that the BEO cannot be traced back to a cold-extracted oil but rather to a distilled or reconstituted bergamot oil. However, it has been shown that the volatile imprint of bergamot constituents can be effectively integrated into lipid foods such as extra virgin olive oil. The feasibility of imparting a citrus bergamot aroma to extra virgin olive oil has been validated.

Fresh bergamot fruits were added to extra virgin olive oil during olive pressing (with concentrations of 10 and 20% w/w) or by infusion of freeze-dried bergamot fruits [113]. From a sensory point of view the aromatization during olive pressing can positively influence sensory quality by covering certain defects, thanks to the increase in citrus, fruity and bitter notes. However, the quantities and quality of bergamots to be added still need to be clearly defined as the aroma of the oil produced with 20% of bergamot exhibited an excessively intense aroma, whereas 10% was more balanced and acceptable. The oil obtained by infusion was found to have an increase in citrus notes, but a smaller perceived sweetness than the previous flavouring technique for sweet and floral.

### 5.3. Incorporation into Functional Foods

BBP, particularly pomace, represent a valuable source of dietary fibre and bioactive compounds. Gattuso et al. [24] analyzed its mineral profile for the first time. Bergamot pomace flour is a significant source of cellulose, hemicellulose and lignin with a great amount of insoluble dietary fibre, associated with beneficial effects on metabolic health and gut microbiota [117].

Bergamot pomace flour has been used to functionalize pasta [24] and biscuits [19]. In these studies, partial substitution of durum wheat flour with bergamot pomace flour improved the nutritional and functional properties of the products, increasing phenolic content and antioxidant activity without significantly compromising technological or sensory characteristics. Notably, antioxidant compounds remained stable even after thermal processing and cooking.

Despite the growing market demand for BJ in beverage formulations and the well-documented functional and health benefits, its application as a food ingredient remains limited, with only a few examples reported in the literature [118].

Another innovative application involved the use of bergamot peel in craft beer production. Carabetta et al. [119] demonstrated that the addition of bergamot peel enriched the phenolic composition of the beverage, improving its nutraceutical value and generating a distinctive chemical fingerprint.

### 5.4. Antimicrobial and Food Preservation Effects

In addition to their nutritional and functional potential, bergamot derivatives have attracted growing scientific interest due to their natural antimicrobial properties and their preservative effects in food products. A previous review [120] reported the inhibitory activity of bergamot flavonoid-rich extracts against several food-related microorganisms, including *Escherichia coli*, *Salmonella enterica*, *Pseudomonas putida*, *Bacillus subtilis*, *Lactococcus lactis*, *Listeria innocua*, *Staphylococcus aureus*, and *Saccharomyces cerevisiae*. In vitro studies also demonstrated inhibitory effects of BJ against *Helicobacter pylori*.

More recent research has explored the antimicrobial potential of bergamot derivatives in real food systems. Lertnirundon and Mahidsanan [118] demonstrated that the incorporation of BJ in Thai steamed pumpkin cake significantly reduced microbial growth, including *Bacillus cereus*, yeast, and mould populations after baking. Similarly, BJ has been investigated as a preservative agent in mozzarella storage liquid, where it reduced the growth of *Pseudomonas* spp. and extended product shelf life [121].

Bergamot pomace extracts have also shown promising effects in minimally processed fruits and vegetables. For example, immersion of fresh-cut artichokes in a flavonoid-enriched BJ extract significantly inhibited the growth of aerobic mesophilic bacteria, Pseudomonas, and Enterobacteriaceae during storage [122].

Further applications include the incorporation of bergamot extracts into edible films and coatings. Chitosan-based films enriched with BJ powder exhibited enhanced antibacterial activity against both Gram-positive bacteria (*Listeria monocytogenes* and *Staphylococcus aureus*) and Gram-negative bacteria (*Escherichia coli* and *Salmonella*) [123].

Similarly, bergamot polyphenol powder incorporated into chitosan coatings improved microbial stability and shelf life of fresh-cut salads [124], reducing microbial growth while maintaining colour, moisture, soluble solids, and sensory properties.

Comparable results were obtained for ready-to-eat strawberries coated with edible films containing bergamot polyphenol extracts and BEO [125]. These coatings significantly delayed microbial spoilage and preserved physicochemical and textural quality during refrigerated storage.

### 5.5. Bergamot-Enriched Beverages

The bioactive richness of bergamot has stimulated growing interest in the development of functional beverages enriched with bergamot-derived ingredients. The chemical and biological characteristics of bergamot components make them attractive candidates for beverage fortification [13].

Carabetta et al. [119] validated a rapid UHPLC–PDA method for the identification and quantification of polyphenols and bitter acids in beers. The study demonstrated that the addition of bergamot significantly increased the phenolic content of the beverages while providing a distinctive nutraceutical and sensory profile.

These findings support the development of novel beverages incorporating bergamot derived ingredients. However, bergamot derivates are rich in oxygen heterocyclic compounds, especially coumarins, furocoumarins, and polymethoxyflavones, which are relevant chemical markers and require safety and regulatory assessment because of their potential adverse effects on human health [43]. Arigò et al. [126] applied advanced LC-MS/MS techniques to quantify coumarins and furocoumarins in bergamot-flavoured beverages comprising juices. Their study showed the presence of these compounds in bergamot-based beverages, such as commercial juices, alcoholic beverages, infusions, and Earl Grey tea, highlighting the need to evaluate their intake.

In addition to compositional profiling [31], BJ has also been tested as a matrix for fermented functional beverages. BJ, fermented with mixed cultures of *Lactobacillus plantarum*, yielded a beverage with probiotics, and favourable sensory properties [127].

The application of microencapsulated bergamot extracts in beverages has also been investigated (Section 5.1) [74] showing their potential for controlled release and stability, highlighting a relevant technological opportunity for the functional beverage sector.

Research so far suggests that ingredients recovered from bergamot can play a valuable role in the development of innovative beverages providing bioactives and improved sensory and functional characteristics.

### 5.6. Outlook

Overall, the available evidence summarized in Table 5 supports the potential use of bergamot-derived bioactive compounds as multifunctional food ingredients, particularly for improving oxidative stability, product quality, and natural preservation strategies. However, their application in real food matrices remains underexplored and less developed than in the nutraceutical and pharmaceutical sectors [128].

The main challenges concern the variability of extract composition, the stability of polyphenols and volatile compounds during processing and storage, their interaction with food matrix components, and possible sensory drawbacks such as bitterness, acidity, astringency, or intense citrus notes. In addition, safety and regulatory aspects require careful consideration, particularly regarding furocoumarins and other oxygen heterocyclic compounds in concentrated extracts or products intended for regular consumption.

Future research should therefore combine formulation studies, shelf-life testing, sensory evaluation, safety assessment, and regulatory analysis in real food systems. Further investigations are also needed in underexplored food categories, such as dairy, meat, and emulsified products, where BBP could provide antioxidant, antimicrobial, textural, or flavouring functions. This integrated approach is essential to support the transition of BBP from underutilized agro-industrial residues to reliable ingredients for industrial food applications.

## 6. Health-Promoting Effects of Bergamot-Derived Bioactives

Bergamot bioactive compounds have attracted increasing attention due to their wide range of biological activities. Growing evidence from preclinical, translational and clinical studies indicates that these compounds may exert beneficial effects through antioxidant, anti-inflammatory and metabolic regulatory mechanisms. Consequently, bergamot has been proposed as a promising source of nutraceutical compounds for the management of oxidative stress, dyslipidaemia, glucose dysregulation, intestinal dysbiosis and neuroinflammation, pathophysiological processes closely involved in the onset and progression of cardiometabolic and neurodegenerative diseases. This section summarizes the pleiotropic biological effects associated with bergamot bioactive compounds (BBC) at the cellular, tissue and systemic levels.

### 6.1. Antioxidant and Cytoprotective Activity

Bergamot is recognized for its marked antioxidant activity, primarily attributable to the high concentration of polyphenols and flavonoids present in both juice and peel [14]. Among the most significant bioactive compounds are glycosylated flavanones such as naringin, neohesperidin, neoeriocitrin, and brutieridin, molecules known for their ability to donate electrons and stabilize reactive oxygen species (ROS) [27].

These phytocompounds exert antioxidant activity through several synergistic mechanisms, including direct free radical neutralization, chelation of pro-oxidant metal ions, and modulation of endogenous antioxidant systems. These activities contribute significantly to the reduction in cellular oxidative stress, a pathophysiological process implicated in ageing and the development of numerous chronic degenerative diseases, including cardiovascular diseases and metabolic disorders [27].

Experimental studies have also demonstrated that BPF are able to reduce lipid peroxidation markers and regulate redox-sensitive signalling pathways, promoting an adaptive cytoprotective response [129]. These properties gives bergamot significant nutraceutical interest, with potential applications in the prevention of systemic oxidative stress and its clinical consequences.

### 6.2. Anti-Inflammatory and Immunomodulatory Effects

Preclinical and translational evidence indicates that various derivatives of *Citrus bergamia* (essential oil, juice/extracts, polyphenolic or flavonoid fractions, leaf extracts and by-products) can attenuate inflammation by modulating redox-sensitive pathways and pro-inflammatory transcriptional programmes, with potential immunomodulatory effects [26,29,39]. In LPS-stimulated THP-1 monocytes, the flavonoid fraction of BJ reduced the inflammatory response via a SIRT1–NF-κB axis, suggesting a direct action on the “set-point” of the innate response [130]. Overall, these findings support bergamot-derived preparations as plausible adjuncts to mitigate low-grade inflammation, but heterogeneity in formulations limits firm conclusions regarding the active constituents and dose–response relationships.

Randomized clinical trials that use standardized products and predefined immuno-inflammatory endpoints remain necessary to define efficacy, optimal dose, and safety in populations at cardiometabolic risk.

### 6.3. Lipid-Lowering and Cardioprotective Properties

The lipid-lowering properties of bergamot in humans are supported by a systematic review in which most included studies reported reductions in total cholesterol, LDL-C, and triglycerides after supplementation with *Citrus bergamia*-based preparations [131]. A meta-analysis of randomized controlled trials also reported average reductions in total cholesterol, LDL-C, and triglycerides, together with an increase in HDL-C, while noting heterogeneity across formulations, background therapies, and clinical settings [132].

Overall, current human evidence suggests that bergamot-derived products may support lipid management, particularly in subjects with cardiometabolic risk [131,132,133,134,135]. However, heterogeneity in formulations, dosage, and study design still limits direct comparison among studies. Further investigations using standardized food-grade preparations are needed to better define efficacy, safety, and applicability in food and nutraceutical products [132].

### 6.4. Glucose Metabolism and Anti-Diabetic Potential

Bergamot is a unique source of flavonoids and polyphenols which, in experimental models, show activity on key pathways of insulin sensitivity and glucose homeostasis (e.g., AMPK, oxidative stress and inflammation), making an antidiabetic effect biologically plausible but not yet definitively proven in clinical settings [136].

In the translation “from bench to bedside”, the most consistent signal remains that of overall cardiometabolic health (especially lipid profile), while the effect on glucose appears more heterogeneous and dependent on phenotype, dose, formulation and the endpoints selected [136,137].

Overall, the available evidence supports the idea that bergamot may help improve an unfavourable metabolic profile, especially when dyslipidaemia and insulin resistance coexist; while its usefulness as a specific anti-diabetic intervention (diabetes prevention or T2D treatment) remains to be defined with larger, longer and better phenotyped studies, including rigorous standardization of the extract and comprehensive reporting of adherence and dietary/pharmacological co-interventions. This broader picture also includes combined formulations such as a supplement containing dry artichoke and bergamot extracts [133], although these combinations increase the difficulty of defining the specific contribution of bergamot. Finally, mechanistic work on antioxidant and cytoprotective effects [138], reinforces biological plausibility for an impact on insulin resistance mediated by oxidative stress, while remaining indirect with respect to clinical glycemic endpoints.

### 6.5. Gut Microbiota Modulation

Growing evidence indicates that bergamot, particularly in the form of polyphenolic extracts or BPF, can significantly modulate the composition and function of the gut microbiota, contributing to the maintenance of intestinal and metabolic homeostasis [117]. Bergamot polyphenols, characterized by partial intestinal bioavailability, largely escape absorption in the upper gastrointestinal tract and reach the colon in native or partially metabolized form, where they come into direct contact with the resident microbial community. Overall, these findings suggest that bergamot may modulate the gut microbiota and contribute to the functional regulation of the intestinal ecosystem, with potential positive effects on dysbiosis and related metabolic and inflammatory alterations.

### 6.6. Neuroprotective and Other Emerging Benefits

Available evidence suggests that bergamot possesses significant neuroprotective properties, primarily attributable to its rich flavonoid and polyphenolic fraction, capable of intervening on multiple pathogenic mechanisms involved in neuronal damage. In vitro models of neurotoxicity showed that bergamot flavonoids have effective protective action against neuronal cells exposed to pro-oxidant stimuli, significantly reducing excessive ROS production and limiting intracellular oxidative stress [139,140]. Treatment with bergamot extracts has been shown to preserve mitochondrial function, preventing alterations in membrane potential and attenuation of apoptosis-related damage, thereby limiting oxidative injury and supporting neuronal survival. Maintenance of mitochondrial integrity translates into a significant improvement in cell viability and survival, suggesting that bergamot flavonoids act not only as direct free radical scavengers but also as modulators of endogenous antioxidant defence systems and adaptive stress responses [132,140].

As previously highlighted, experimental studies conducted on neuronal and cells showed that treatment with bergamot extracts attenuated oxidative stress, cytotoxicity, improving cell viability and limiting cell death [141]. In particular, the flavonoids and polyphenols contained in bergamot appear to modulate the cascade of intracellular events leading to apoptosis, including stabilizing mitochondrial membrane potential, reducing the release of cytochrome c into the cytosol, and the consequent inhibition of caspase-3 and -9 activation. These effects contribute to preserving mitochondrial function and maintaining the structural integrity of nerve cells.

In addition to the properties just listed, bergamot displays a series of emerging benefits that are attracting growing scientific interest. One emerging aspect concerns autophagy and cellular remodelling. Certain bergamot flavonoids have been associated with the stimulation of lipid autophagy (lipophagy) in animal models of fatty liver disease, a process that promotes the selective degradation of lipid deposits and potentially contributes to cellular detoxification and the prevention of toxic lipid accumulation (mechanisms of bergamot-induced lipophagy) [142]. Still in the emerging field of musculoskeletal health, bergamot extracts have been evaluated for their potential effect in improving muscle mass and function, suggesting a possible role in the management of conditions such as osteosarcopenia and age-related obesity, with benefits on contractile capacity and resistance to muscle oxidative stress [143].

### 6.7. Summary and Implications

Bioactive compounds derived from bergamot, particularly its characteristic polyphenolic flavanones, show multiple biological activities that include antioxidant cytoprotection, anti-inflammatory modulation, improved lipid/glycemic to reduce waste of food processing meostasis, rebalancing of the gut microbiota, and emerging neuroprotective/musculoskeletal benefits, as demonstrated by integrated preclinical and clinical data. Table 6 summarizes these actions, revealing a mechanistic convergence on Nrf2/NF-κB pathways, mitochondrial conservation, and immunometabolic axes, with the strongest human validation in populations at risk of dyslipidaemia/cardiometabolism (e.g., LDL-C ↓, hs-CRP ↓ via RCT/meta-analysis) compared to exploratory preclinical signals in microbiota/neuroprotection [132]. Despite promising multi-target potential, heterogeneity in formulations (BPF, juice, phytosomes), dosing, and populations limits clarity and attribution of dose response, underscoring the need for standardized extracts, adequately powered RCTs with immuno-inflammatory/intestinal endpoints, long-term safety data, and direct comparative studies against guideline recommended therapies. With the advancement of sustainable valorisation of by-products (e.g., peel polyphenols), bergamot is positioned as a valuable adjuvant in Mediterranean-style precision nutraceuticals for chronic oxidative/inflammatory conditions, justifying investment in translational phenotyping for personalized applications at the cardiometabolic, hepatic and age-related levels [26,143].

## 7. Conclusions and Future Perspectives

Bergamot (*Citrus bergamia*) is a distinctive citrus species mainly associated with the province of Reggio Calabria in Southern Italy, where specific pedoclimatic conditions contribute to its characteristic chemical and aromatic profile. Although bergamot is globally recognized for its essential oil, increasing attention is now being directed toward the valorisation of the whole fruit and the by-products generated during processing. Future research should also consider how changing climatic conditions may affect bergamot cultivation, fruit quality, bioactive composition, and by-product availability. BBP, particularly peel, pomace, and other solid fractions, represent valuable sources of polyphenols, flavonoids, pectins, dietary fibres, volatile compounds, and other bioactive molecules. Their recovery through green extraction technologies and their stabilization through suitable formulation strategies may support their use as functional ingredients in food systems.

However, several gaps still limit their practical application. Future studies should prioritize scalable and economically feasible extraction processes, supported by techno-economic assessments and life-cycle evaluation. Greater attention is also needed to improve the stability of bergamot-derived bioactives during processing and storage, especially when extracts are incorporated into real food matrices under industrial conditions.

From an application perspective, research should move beyond extract characterization and focus on formulation performance, sensory acceptance, shelf-life extension, and interactions with food matrix components. Promising directions include natural preservatives, antioxidant ingredients, active or bio-based packaging systems, and applications in underexplored food categories such as dairy, meat, and emulsified products.

Safety and regulatory aspects also require further investigation, particularly regarding furocoumarins and other oxygen heterocyclic compounds naturally present in citrus matrices. Addressing bioavailability, dosage, safety, and regulatory feasibility will be essential to support consumer protection, health-related claims, and industrial adoption.

Overall, BBP offer a promising opportunity for the development of value-added food ingredients. Their successful valorisation will depend on integrated approaches combining extraction optimization, stabilization, food formulation, safety assessment, and industrial validation.

## Figures and Tables

**Table 1:** Bergamot by-products: origin, bioactive compounds, and functional relevance.

By-Product Fraction	Origin/Processing Stage	Major Bioactive Compounds	References
Peel (flavedo + albedo)	Residue after cold-pressing for essential oil extraction	Flavanone glycosides, pectins, phenolic acids, residual terpenes	[22,23]
Pomace	Solid residue from bergamot processing, locally known as “pastazzo” (peel + pulp + seeds)	Polyphenols, flavonoids, dietary fibres, residual essential oil compounds	[19,24]
Seeds	By-product from juice/pomace processing	Limonoids (limonin, nomilin, glucosides), minor phenolics	[25]
Peel residues (post-extraction)	Residual biomass after essential oil recovery	Residual monoterpenes (limonene, linalool, linalyl acetate), phenolics, polysaccharides	[13]
Pomace-derived flour	Dried and milled pomace	Fibres, minerals, polyphenols	[19,24]
Peel, seeds, leaves, juice, essential oil	Combined industrial residues	Polyphenols, flavonoids, phenolic derivatives, minor bioactive constituents	[26]

**Table 2:** Correlation between major phytochemical classes of bergamot, structural features, and biological activities (2023–2025).

Phytochemical Class	Representative Compounds	Key Structural Features	Main Molecular Targets/Mechanisms	References
Flavanone glycosides	Neoeriocitrin, naringin, neohesperidin, eriocitrin	O- and C-glycosylation; catechol and methoxy groups; high polarity	ROS scavenging; metal chelation; activation of Nrf2; inhibition of NF-κB	[10,14]
HMG-flavanones	Brutieridin, melitidin	3-hydroxy-3-methylglutaryl (HMG) moiety; statin-like pharmacophore	Competitive inhibition of HMG-CoA reductase; modulation of lipid metabolism	[8,42]
Phenolic acids	Caffeic, ferulic, p-coumaric, sinapic, chlorogenic acids	Hydroxycinnamic backbone; conjugated double bonds; free hydroxyl groups	Hydrogen atom transfer (HAT); inhibition of lipid peroxidation; metal chelation	[29,31].
Limonoids	Limonin, nomilin	Tetranortriterpenoid skeleton; lactone and epoxide groups	Activation of Nrf2/HO-1; suppression of NF-κB and MAPK	[26,34,35]
Coumarins & furanocoumarins	Bergapten, bergamottin, citropten	Benzopyrone core; furan ring (furanocoumarins)	Interaction with CYP450 enzymes (CYP3A4); antimicrobial mechanisms	[36,43]
Alkaloids	Synephrine, indole derivatives	Nitrogen-containing heterocycles; polar structures	Modulation of adrenergic and cellular signalling pathways	[37]
Volatile terpenoids	Limonene, linalool, linalyl acetate	Mono- and oxygenated monoterpenes; high volatility	Membrane disruption in microorganisms; antioxidant interactions	[39]

**Table 3:** Green extraction technologies applied to bergamot by-products (2020–2025).

Technology	Solvent System	Target Compounds	Main Advantages	Main Limitations	Food-Grade Suitability	References
CET	Organic solvents, water, steam	Polyphenols, pectins, oils	Mature and scalable technology	High solvent/energy use, safety concerns	Variable (depends on solvent)	[50,51]
UAE	Water, ethanol, hydroalcoholic mixtures	Polyphenols, flavonoids	Short time; mild conditions; reduced solvent use	Possible degradation at high intensity; scale-up challenges	High	[12,53,54,55,56]
MAE	Water, ethanol, hydroalcoholic mixtures	Polyphenols, pectins	Rapid extraction; improved yield; reduced solvent use	Risk of overheating; non-uniform heating	High	[12,59,60,62]
EAE	Aqueous enzyme systems	Polyphenols, pectins	High selectivity; mild conditions; improved matrix permeability	Long processing time; enzyme cost; process sensitivity	Very high	[21,61,63]
SFE	CO_2_ ± ethanol	Essential oils, lipophilic compounds	Solvent-free extracts; high selectivity; mild temperatures	High capital cost; low efficiency for polar compounds	High	[50,59,64]
NADES	Choline chloride-based mixtures, organic acids, sugars	Polyphenols, antioxidants	Tunable polarity; high solubilization capacity; low volatility	High viscosity; mass transfer limit; regulatory uncertainty	Potential	[63,65,66,67,68,69,70]
Hybrid/Integrated Processes	Combined systems	Multi-fraction recovery	Improved selectivity; reduced solvent use; multi-product valorisation	Process complexity; optimization required	High	[61,71]

**Table 5:** Food applications of bergamot-derived ingredients (2020–2025).

Application Area	Main Technological Function	Food Model/Application	References
Antioxidant & Functional properties	Radical scavenging activity, phenolic enrichment, oxidative stability improvement, protection during heat processing	Vegetable fat, biscuits, vegetable oils, apple juice	[20,23,74,113]
Food Preservation & Shelf-Life Extension	Microbial growth inhibition; lipid oxidation control; preservation of texture and sensory quality	Thai steamed pumpkin cake, mozzarella preserving liquid, fresh-cut artichokes, chitosan edible films	[121,122,123,124]
Antimicrobial Effects	Inhibition of foodborne bacteria, yeasts, and moulds	Biscuits, mozzarella, fresh-cut product	[121,122,123]
Flavouring & Aromatization	Citrus and floral notes, modulation of oil sensory profile	Bergamot-flavoured beer, olive oil, sensory evaluation studies	[114,115,116]
Enriched Beverages	Phenolic and bitter acid enrichment, probiotic potential, antioxidant activity enhanced	Beer, fermented bergamot juice, apple juice enriched with microencapsulated bergamot extract	[74,119,127]
Food Functionalisation	Improving phenolic profile, and functional value	Pasta, biscuits, functional beverages	[19,24]

**Table 6:** Health-promoting effects and mechanisms of bergamot-derived bioactives (2020–2025).

Effect Category	Key Mechanisms	Models/Level of Evidence	References
Antioxidant & Cytoprotective	ROS scavenging, metal chelation, Nrf2 activation, reduced lipid peroxidation, mitochondrial protection	Mainly in vitro and animal models	[140,144]
Anti-inflammatory & Immunomodulatory	NF-κB inhibition, SIRT1 activation, cytokine modulation, reduced low-grade inflammation	Preclinical & translational studies	[26,29,145]
Lipid-Lowering & Cardioprotective	Reduced TC, LDL-C, TG, hs-CRP; increased HDL-C; improved endothelial function	Human RCTs, meta-analyses; strongest in dyslipidaemia/cardiometabolic risk	[131,133]
Glucose Metabolism & Anti-Diabetic	AMPK signalling, reduced oxidative stress, insulin sensitivity modulation, postprandial response effects	Human studies with mixed results; stronger relevance in insulin-resistant phenotypes	[137,138,139,140,141,142,143,144,145,146]
Gut Microbiota Modulation	Increased beneficial bacteria, SCFA production, barrier integrity; reduced LPS-related inflammation	Mainly animal models of dysbiosis or high-fat diet	[147]
Neuroprotective & Emerging	Reduced apoptosis, mitochondrial protection, Nrf2-related defences, lipophagy, reduced muscle oxidative stress	Exploratory evidence, mainly in vitro and animal models	[138,140,143]

Health benefits, proposed mechanisms and level of evidence of bioactive compounds derived from bergamot (2020–2025). Mechanistic pathways are mainly derived from in vitro and animal models, while findings related to lipid reduction and cardiometabolic metabolism are supported by randomized controlled trials and meta-analyses in at-risk populations. Emerging effects (e.g., microbiota modulation, neuroprotection, lipophagy, and musculoskeletal outcomes) are predominantly preclinical and should be interpreted as biologically plausible but not yet clinically established.

## Data Availability

No new data were created or analyzed in this study. Data sharing is not applicable to this article.

## Abbreviations

The following abbreviations are used in this manuscript:

BBP	Bergamot by-products
NADES	Natural deep eutectic solvents
BPF	Bergamot Polyphenolic Fraction
BLPF	Bergamot leaf polyphenol fractions
BFPF	Bergamot fruit polyphenol fractions
HMG	3-hydroxy-3-methylglutaryl
BJ	Bergamot juice
HPLC	High-performance liquid chromatography
TPC	Total polyphenolic content
BEO	Bergamot essential oil
CYP	cytochrome P450 enzyme
HAT	Hydrogen atom transfer
CET	Conventional extraction techniques
UAE	Ultrasound-Assisted Extraction
MAE	Microwave-Assisted Extraction
EAE	Enzyme-Assisted Extraction
SFE	Supercritical Fluid Extraction
DES	Deep eutectic solvents
BBC	Bergamot bioactive compounds
OSA	Octenyl succinic anhydride
SAS	Supercritical anti-solvent
PGSS	Particles from gas-saturated solutions
PIT	Phase inversion temperature
SLN	Solid lipid nanoparticles
NLC	Nanostructured lipid carriers
MLV	Multilamellar vesicles
SUV	Small unilamellar vesicles
LUV	Large unilamellar vesicles
ROS	Reactive oxygen species
SOD	Superoxide dismutase

## References

[1] Radha A., Ahluwalia V., Rai A.K., Varjani S., Awasthi M.K., Sindhu R., Binod P., Saran S., Kumar V. (2024). The way forward to produce nutraceuticals from agri-food processing residues: Obstacle, solution, and possibility. J. Food Sci. Technol..

[2] Lizárraga-Chaidez M., Abadía-García L., Mendoza-Sánchez M.J., Huerta-Manzanilla E.L., Mendoza-Sánchez M. (2024). Optimization of the green extraction process of antioxidants derived from grape pomace. Sustain. Chem. Pharm..

[3] Fernandes F.A., Heleno S.A., Pinela J., Carocho M., Prieto M.A., Ferreira I.C.F.R., Barros L. (2022). Recovery of Citric Acid from Citrus Peels: Ultrasound-Assisted Extraction Optimized by Response Surface Methodology. Chemosensors.

[4] Fernández-Cabal J., Avilés-Betanzos K.A., Cauich-Rodríguez J.V., Ramírez-Sucre M.O., Rodríguez-Buenfil I.M. (2025). Recent Developments in Citrus aurantium L.: An Overview of Bioactive Compounds, Extraction Techniques, and Technological Applications. Processes.

[5] Sanli I., Ozkan G., Şahin-Yeşilçubuk N. (2025). Green extractions of bioactive compounds from citrus peels and their applications in the food industry. Food Res. Intern..

[6] AlZahabi S., Mamdouh W. (2025). Valorization of citrus processing waste into high-performance bionanomaterials: Green synthesis, biomedicine, and environmental remediation. RSC Adv..

[7] Di Donna L., De Luca G., Mazzotti F., Napoli A., Salerno R., Taverna D., Sindona G. (2009). Statin-like Principles of Bergamot Fruit (*Citrus bergamia*): Isolation of 3-Hydroxymethylglutaryl Flavonoid Glycosides. J. Nat. Prod..

[8] Huang Y., Tocmo R., Nauman M.C., Haughan M.A., Johnson J.J. (2021). Defining the Cholesterol Lowering Mechanism of Bergamot (*Citrus bergamia*) Extract in HepG2 and Caco-2 Cells. Nutrients.

[9] Maiuolo J., Bulotta R.M., Ruga S., Nucera S., Macrì R., Scarano F., Oppedisano F., Carresi C., Gliozzi M., Musolino V. (2024). The Postbiotic Properties of Butyrate in the Modulation of the Gut Microbiota: The Potential of Its Combination with Polyphenols and Dietary Fibers. Int. J. Mol. Sci..

[10] Baron G., Altomare A., Mol M., Garcia J.L., Correa C., Raucci A., Mancinelli L., Mazzotta S., Fumagalli L., Trunfio G. (2021). Analytical Profile and Antioxidant and Anti-Inflammatory Activities of the Enriched Polyphenol Fractions Isolated from Bergamot Fruit and Leave. Antioxidants.

[11] Ferrarese I., Lupo M.G., Rossi I., Sut S., Loschi F., Allegrini P., Riva A., Ferri N., Dall’Acqua S. (2023). Bergamot (*Citrus bergamia*) Peel Extract as New Hypocholesterolemic Agent Modulating PCSK9 Expression. J. Funct. Foods.

[12] Gattuso A., Piscopo A., Romeo R., De Bruno A., Poiana M. (2023). Recovery of Bioactive Compounds from Calabrian Bergamot Citrus Waste: Selection of Best Green Extraction. Agriculture.

[13] Della Vedova L., Gado F., Vieira T.A., Grandini N.A., Palácio T.L.N., Siqueira J.S., Carini M., Bombardelli E., Correa C.R., Aldini G. (2023). Chemical, Nutritional and Biological Evaluation of a Sustainable and Scalable Complex of Phytochemicals from Bergamot By-Products. Molecules.

[14] Demircan B., Velioglu Y.S., Giuffrè A.M. (2023). Bergamot Juice Powder with High Bioactive Properties: Spray-Drying for the Preservation of Antioxidant Activity and Ultrasound-Assisted Extraction for Enhanced Phenolic Compound Extraction. J. Food Sci..

[15] Sabanci S., Cevik M., Göksu A. (2021). Investigation of Time Effect on Pectin Production from Citrus Wastes with Ohmic Heating-Assisted Extraction Process. J. Food Process Eng..

[16] Petrotos K., Giavasis I., Gerasopoulos K., Mitsagga C., Papaioannou C., Gkoutsidis P. (2021). Optimization of Vacuum-Microwave-Assisted Extraction of Natural Polyphenols and Flavonoids from Raw Solid Waste of the Orange Juice Producing Industry at Industrial Scale. Molecules.

[17] Phucharoenrak P., Muangnoi C., Trachootham D. (2022). A Green Extraction Method to Achieve the Highest Yield of Limonin and Hesperidin from Lime Peel Powder (*Citrus aurantifolia*). Molecules.

[18] Plaza M., Marina M.L. (2025). Natural Deep Eutectic Solvents and Ultrasound-Assisted Extraction for the Recovery of Antioxidant Phenolic Compounds from Orange Pomace. Microchem. J..

[19] Laganà V., Giuffrè A.M., De Bruno A., Poiana M. (2022). Formulation of Biscuits Fortified with a Flour Obtained from Bergamot By-Products (*Citrus bergamia*, Risso). Foods.

[20] Gattuso A., Piscopo A., Santacaterina S., Imeneo E., De Bruno A., Poiana M. (2023). Fortification of Vegetable Fat with Natural Antioxidants Recovered from Bergamot Pomace for Use as an Ingredient for the Production of Biscuits. Sustain. Food Technol..

[21] Serra M., Macrì R., Bonacci S., Ritorto G., Ussia S., Nucera S., Caminiti R., Ruga S., Altomare C., Tucci L. (2025). The Second Life of *Citrus bergamia*: Bioavailability Analysis of a New Formulation Using Waste-Based Microencapsulation as a Valuable Source of Bioactive Compounds. Pharmac. Rep..

[22] Mandalari G., Bennett R.N., Bisignano G., Saija A., Dugo G., Lo Curto R.B., Faulds C.B., Waldron K.W. (2006). Characterization of flavonoids and pectins from bergamot (*Citrus bergamia* Risso) peel, a major byproduct of essential oil extraction. J. Agric. Food Chem..

[23] Siano F., Picariello G., Castaldo D., Cautela D., Caruso T., Vasca E. (2023). Monitoring antioxidants by coulometry: Quantitative assessment of the strikingly high antioxidant capacity of bergamot (*Citrus bergamia* R.) by-products. Talanta.

[24] Gattuso A., De Bruno A., Piscopo A., Santacaterina S., Frutos M.J., Poiana M. (2024). Bergamot Pomace Flour: From Byproduct to Bioactive Ingredient for Pasta Production. Sustainability.

[25] Russo M., Arigò A., Calabrò M.L., Farnetti S., Mondello L., Dugo P. (2016). Bergamot (*Citrus bergamia* Risso) as a source of nutraceuticals: Limonoids and flavonoids. J. Funct. Foods.

[26] Russo C., Lombardo G.E., Bruschetta G., Rapisarda A., Maugeri A., Navarra M. (2024). Bergamot Byproducts: A Sustainable Source to Counteract Inflammation. Nutrients.

[27] Nauman M.C., Johnson J.J. (2019). Clinical Application of Bergamot (*Citrus bergamia*) for Reducing High Cholesterol and Cardiovascular Disease Markers. Integr. Food Nutr. Metab..

[28] Da Pozzo E., De Leo M., Faraone I., Milella L., Cavallini C., Piragine E., Testai L., Calderone V., Pistelli L., Braca A. (2018). Antioxidant and Antisenescence Effects of Bergamot Juice. Oxidative Med. Cell. Longev..

[29] Adorisio S., Muscari I., Fierabracci A., Thi Thuy T., Marchetti M.C., Ayroldi E., Delfino D.V. (2023). Biological effects of bergamot and its potential therapeutic use as an anti-inflammatory, antioxidant, and anticancer agent. Pharm. Biol..

[30] Carullo G., Ramunno A., Sommella E.M., De Luca M., Belsito E.L., Frattaruolo L., Brindisi M., Campiglia P., Cappello A.R., Aiello F. (2022). Ultrasound-Assisted Extraction, Chemical Characterization, and Impact on Cell Viability of Food Wastes Derived from Southern Italy Autochthonous Citrus Fruits. Antioxidants.

[31] Gattuso A., Mafrica R., Cannavò S., Mafrica D., De Bruno A., Poiana M. (2024). Quality Evaluation of Bergamot Juice Produced in Different Areas of Calabria Region. Foods.

[32] Vieira T.A., Seloto D.G., Rizzi J.S., Peixoto P.V.L., Corrêa G.V.B., Siqueira J.S., Grandini N.A., Nakandakare-Maia E.T., Valente L.C., Francisqueti-Ferron F.V. (2025). Bergamot Leaf Extract as an Agent Against Chronic Liver Diseases? In Vitro and In Vivo Findings on Oxidative Stress Modulation. Antioxidants.

[33] Giuffrè A.M. (2019). Bergamot (*Citrus bergamia*, Risso): The Effects of Cultivar and Harvest Date on Functional Properties of Juice and Cloudy Juice. Antioxidants.

[34] Impellitteri F., Multisanti C.R., Riolo K., Zicarelli G., Porretti M., Cafeo G., Russo M., Dugo P., Di Bella G., Piccione G. (2025). Bergamot (*Citrus bergamia*): A Potential New Nutraceutical Against Cellular and Physiological Alterations Induced by Emerging Contaminants in Sentinel Organisms. Antioxidants.

[35] Saini R.K., Ranjit A., Sharma K., Prasad P., Shang X., Gowda K.G.M., Keum Y.-S. (2022). Bioactive Compounds of Citrus Fruits: A Review of Composition and Health Benefits of Carotenoids, Flavonoids, Limonoids, and Terpenes. Antioxidants.

[36] Gardana C., Nalin F., Simonetti P. (2008). Evaluation of Flavonoids and Furanocoumarins from *Citrus bergamia* (Bergamot) Juice and Identification of New Compounds. Molecules.

[37] Divyasakthi M., Sarayu Y.C.L., Shanmugam D.K., Karthigadevi G., Subbaiya R., Karmegam N., Kaaviya J.J., Chung W.J., Chang S.W., Ravindran B. (2025). A Review on Innovative Biotechnological Approaches for the Upcycling of Citrus Fruit Waste to Obtain Value-Added Bioproducts. Food Technol. Biotechnol..

[38] Cautela D., Vella F.M., Laratta B. (2019). The Effect of Processing Methods on Phytochemical Composition in Bergamot Juice. Foods.

[39] Perna S., Spadaccini D., Botteri L., Girometta C., Riva A., Allegrini P., Petrangolini G., Infantino V., Rondanelli M. (2019). Efficacy of bergamot: From anti-inflammatory and anti-oxidative mechanisms to clinical applications as preventive agent for cardiovascular morbidity, skin diseases, and mood alterations. Food Sci. Nutr..

[40] Azmir J., Zaidul I.S.M., Rahman M.M., Sharif K.M., Mohamed A., Sahena F., Jahurul M.H.A., Ghafoor K., Norulaini N.A.N., Omar A.K.M. (2013). Techniques for extraction of bioactive compounds from plant materials: A review. J. Food Eng..

[41] Lario Y., Sendra E., García-Pérez J., Fuentes C., Sayas-Barberá E., Fernández-López J., Pérez-Álvarez J.A. (2004). Preparation of high dietary fiber powder from lemon juice by-products. Innov. Food Sci. Emerg. Technol..

[42] Yang Y., Trevethan M., Wang S., Zhao L. (2022). Beneficial Effects of Citrus Flavanones Naringin and Naringenin and Their Food Sources on Lipid Metabolism: An Update on Bioavailability, Pharmacokinetics, and Mechanisms. J. Nutr. Biochem..

[43] Cafeo G., Russo M., Mondello L., Dugo P. (2024). Quantitative Analysis of Oxygen Heterocyclic Compounds Using Liquid Chromatography Coupled to Tandem Mass Spectrometry: Method Development and Environmental Assessment. J. Food Compos. Anal..

[44] Carpentieri S., Soltanipour F., Ferrari G., Pataro G., Donsì F. (2021). Emerging Green Techniques for the Extraction of Antioxidants from Agri-Food By-Products as Promising Ingredients for the Food Industry. Antioxidants.

[45] Ademosun A.O. (2022). Citrus Peels Odyssey: From the Waste Bin to the Lab Bench to the Dining Table. Appl. Food Res..

[46] Saini A., Panesar P.S. (2020). Beneficiation of Food Processing By-Products through Extraction of Bioactive Compounds Using Neoteric Solvents: A Review. LWT.

[47] Singh B., Singh J.P., Kaur A., Singh N. (2020). Phenolic Composition, Antioxidant Potential and Health Benefits of Citrus Peel. Food Res. Inter..

[48] Andrade M.A., Barbosa C.H., Shah M.A., Ahmad N., Vilarinho F., Khwaldia K., Silva A.S., Ramos F. (2023). Citrus By-Products: Valuable Source of Bioactive Compounds for Food Applications. Antioxidants.

[49] Mahato N., Sharma K., Sinha M., Cho M.H. (2018). Citrus Waste Derived Nutra-/Pharmaceuticals for Health Benefits: Current Trends and Future Perspectives. J. Funct. Foods.

[50] Prado J.M., Vardanega R., Debien I.C.N., Meireles M.A.A., Gerschenson L.N., Sowbhagya H.B., Chemat S., Galanakis C.M. (2021). Conventional Extraction. Food Waste Recovery: Processing Technologies and Industrial Techniques.

[51] Sagar N.A., Pareek S., Sharma S., Yahia E.M., Lobo M.G. (2018). Fruit and Vegetable Waste: Bioactive Compounds, Their Extraction, and Possible Utilization. Compr. Rev. Food Sci. Food Saf..

[52] Chemat F., Abert Vian M., Ravi H.K., Khadhraoui B., Hilali S., Perino S., Tixier A.S.F. (2019). Review of Alternative Solvents for Green Extraction of Food and Natural Products: Panorama, Principles, Applications and Prospects. Molecules.

[53] Nie J., Chen D., Ye J., Lu Y., Dai Z. (2021). Optimization and Kinetic Modeling of Ultrasonic-Assisted Extraction of Fucoxanthin from Edible Brown Algae Sargassum fusiforme Using Green Solvents. Ultrason. Sonochem..

[54] Hadidi M., Ibarz A., Pagan J. (2020). Optimisation and Kinetic Study of the Ultrasonic-Assisted Extraction of Total Saponins from Alfalfa (*Medicago sativa*) and Its Bioaccessibility Using the Response Surface Methodology. Food Chem..

[55] Fu X., Wang D., Belwal T., Xie J., Xu Y., Li L., Zou L., Zhang L., Luo Z. (2021). Natural Deep Eutectic Solvent Enhanced Pulse-Ultrasonication Assisted Extraction as a Multi-Stability Protective and Efficient Green Strategy to Extract Anthocyanin from Blueberry Pomace. LWT-Food Sci. Technol..

[56] Yusoff I.M., Mat Taher Z., Rahmat Z., Chua L.S. (2022). A Review of Ultrasound-Assisted Extraction for Plant Bioactive Compounds: Phenolics, Flavonoids, Thymols, Saponins and Proteins. Food Res. Int..

[57] Cui Q., Wang L., Wang G., Zhang A., Wang X., Jiang L. (2021). Ultrasonication Effects on Physicochemical and Emulsifying Properties of Cyperus esculentus (Tiger Nut) Seed Proteins. LWT-Food Sci. Technol..

[58] Xuereb M.A., Psakis G., Attard K., Lia F., Gatt R. (2025). A Comprehensive Analysis of Non-Thermal Ultrasonic-Assisted Extraction of Bioactive Compounds from Citrus Peel Waste Through a One-Factor-at-a-Time Approach. Molecules.

[59] Anticona M., Blesa J., Frigola A., Esteve M.J. (2020). High Biological Value Compounds Extraction from Citrus Waste with Non-Conventional Methods. Foods.

[60] Agregán R., Munekata P.E.S., Feng X., Astray G., Gullón B., Lorenzo J.M. (2021). Recent Advances in the Extraction of Polyphenols from Eggplant and Their Application in Foods. LWT-Food Sci. Technol..

[61] Díaz-de-Cerio E., Trigueros E. (2025). Evaluating the Sustainability of Emerging Extraction Technologies for Valorization of Food Waste: Microwave, Ultrasound, Enzyme-Assisted, and Supercritical Fluid Extraction. Agriculture.

[62] Zhao Q., Xiong H., Selomulya C., Chen X.D., Zhong H., Wang S., Sun W., Zhou Q. (2012). Enzymatic Hydrolysis of Rice Dreg Protein: Effects of Enzyme Type on the Functional Properties and Antioxidant Activities of Recovered Proteins. Food Chem..

[63] Putnik P., Bursać Kovačević D., Režek Jambrak A., Barba F.J., Cravotto G., Binello A., Lorenzo J.M., Shpigelman A. (2017). Innovative “Green” and Novel Strategies for the Extraction of Bioactive Added Value Compounds from Citrus Wastes—A Review. Molecules.

[64] González-Miquel M., Díaz I. (2019). Valorization of Citrus Waste through Sustainable Extraction Processes. Food Industry Wastes.

[65] Santana-Mayor Á., Rodríguez-Ramos R., Herrera-Herrera A.V., Socas-Rodríguez B., Rodríguez-Delgado M.Á. (2021). Deep eutectic solvents: The new generation of green solvents in analytical chemistry. TrAC Trends Anal. Chem..

[66] Socas-Rodríguez B., Mendiola J.A., Rodríguez-Delgado M.Á., Ibáñez E., Cifuentes A. (2022). Safety assessment of citrus and olive by-products using a sustainable methodology based on natural deep eutectic solvents. J. Chromatogr. A.

[67] Mišan A., Nađpal J., Stupar A., Pojić M., Mandić A., Verpoorte R., Choi Y.H. (2020). The perspectives of natural deep eutectic solvents in agri-food sector. Crit. Rev. Food Sci. Nutr..

[68] Chen J., Li Y., Wang X., Liu W. (2019). Application of Deep Eutectic Solvents in Food Analysis: A Review. Molecules.

[69] Torres-Valenzuela L.S., Ballesteros-Gómez A.M., Rubio S. (2020). Green solvents for the extraction of high added-value compounds from agri-food waste. Food Eng. Rev..

[70] Gomez-Urios C., Siroli L., Grassi S. (2025). Sustainable valorization of citrus by-products: Natural deep eutectic solvents for bioactive extraction and biological applications of Citrus sinensis peel. Eur. Food Res. Technol..

[71] Clarke C.J., Tu W.-C., Levers O., Bröhl A., Hallett J.P. (2018). Green and sustainable solvents in chemical processes. Chem. Rev..

[72] Mehta N., Kumar P., Verma A.K., Umaraw P., Kumar Y., Malav O.P., Sazili A.Q., Domínguez R., Lorenzo J.M. (2022). Microencapsulation as a Noble Technique for the Application of Bioactive Compounds in the Food Industry: A Comprehensive Review. Appl. Sci..

[73] Stabrauskiene J., Pudziuvelyte L., Bernatoniene J. (2024). Optimizing Encapsulation: Comparative Analysis of Spray-Drying and Freeze-Drying for Sustainable Recovery of Bioactive Compounds from *Citrus* x *paradisi* L. Peels. Pharmaceuticals.

[74] Gattuso A., Giacondino C., Santacaterina S., Piscopo A., De Bruno A. (2025). Freeze-dried microencapsulation of bergamot pomace extract: Stability and antioxidant performance in hydrophilic and lipophilic systems. Sustain. Food Technol..

[75] Başyiğit B., Yücetepe M., Akyar G., Karaaslan A., Karaaslan M. (2022). Enhancing thermal and emulsifying resilience of pomegranate protein with gum Arabic conjugation. Colloids Surf. B Biointerfaces.

[76] Nhouchi Z., Watuzola R., Pensé-Lhéritier A.M. (2022). A review on octenyl succinic anhydride modified starch-based Pickering-emulsion: Instabilities and ingredients interactions. J. Texture Stud..

[77] Fernandes B., Oliveira M.C., Marques A.C., dos Santos R.G., Serrano C. (2024). Microencapsulation of Essential Oils and Oleoresins: Applications in Food Products. Foods.

[78] Muhoza B., Uriho A. (2025). Freeze-dried essential oils encapsulated in biopolymeric matrices: Design, formulation, and stability: A comprehensive review. Food Biophys..

[79] Kandasamy S., Naveen R. (2022). A review on the encapsulation of bioactive components using spray-drying and freeze-drying techniques. J. Food Process Eng..

[80] Alrosan M., Al-Rabadi N., Alu’datt M.H., Al-Qaisi A., Al-Shunnaq E.E., Abu-Khalaf N., Maghaydah S., Assaf T., Hidmi T., Tan T.-C. (2025). Complex coacervation of plant-based proteins and polysaccharides: Sustainable encapsulation techniques for bioactive compounds. Food Eng. Rev..

[81] Napiórkowska A., Kurek M. (2022). Coacervation as a Novel Method of Microencapsulation of Essential Oils—A Review. Molecules.

[82] Patel P.R., Haemmerich D. (2024). Review on electrospray nanoparticles for drug delivery: Exploring applications. Polym. Adv. Technol..

[83] Munteanu B.S., Vasile C. (2021). Encapsulation of Natural Bioactive Compounds by Electrospinning—Applications in Food Storage and Safety. Polymers.

[84] Pala Avcı N., Aral Yılmaz N., Nergis F.B. (2024). The effect of essential oil on fiber morphology and surface properties in coaxial nanofibers. Uludag Univ. J. Fac. Eng..

[85] Klettenhammer S., Ferrentino G., Morozova K., Scampicchio M. (2020). Novel Technologies Based on Supercritical Fluids for the Encapsulation of Food Grade Bioactive Compounds. Foods.

[86] Wong S.T.S., Kamari A., Abdullah N.N.A., Yusof N., Sutapa I.W. (2024). Preparation and characterization of bergamot essential oil nanoemulsion. IOP Conf. Ser. Earth Environ. Sci..

[87] Safaya M., Rotliwala Y.C. (2020). Nanoemulsions: A review on low energy formulation methods, characterization, applications and optimization technique. Mater. Today Proc..

[88] Liu Q., Huang H., Chen H., Lin J., Wang Q. (2019). Food-Grade Nanoemulsions: Preparation, Stability and Application in Encapsulation of Bioactive Compounds. Molecules.

[89] Volonté P., Ricci C., Arnoldi S., Roda G., Del Favero E., Puoci F., Minghetti P., Franzè S., Cilurzo F. (2025). Bergamot waste derived lipid nanosystems as novel excipients for (trans)dermal drug delivery. J. Drug Deliv. Sci. Technol..

[90] Davodabadi F., Nasri N., Valizadeh N., Haji Ali B., Ghotekar S., Sargazi S., Barani M., Rahman M.M. (2025). Nanotechnology-enhanced delivery systems for bioactive citrus compounds: A comprehensive review. Crit. Rev. Food Sci. Nutr..

[91] Ashfaq R., Rasul A., Asghar S., Kovács A., Berkó S., Budai-Szűcs M. (2023). Lipid Nanoparticles: An Effective Tool to Improve the Bioavailability of Nutraceuticals. Int. J. Mol. Sci..

[92] Pawar S.D., Gawali K., Jat S., Singh P., Datusalia A.K., Kulhari H., Kumar P. (2024). Physiochemical characterization and pharmacokinetic assessment of Bergamottin solid lipid nanoparticles. J. Drug Deliv. Sci. Technol..

[93] Maiti T.K., Parvate S., Dixit P., Singh J., Reddy V.J., Bhuvanesh E., Chattopadhyay S. (2023). Liposome for encapsulation of essential oil and fatty acids. Liposomal Encapsulation in Food Science and Technology.

[94] Dehnad D., Emadzadeh B., Ghorani B., Rajabzadeh G., Kharazmi M.S., Jafari S.M. (2022). Nano-vesicular carriers for bioactive compounds and their applications in food formulations. Crit. Rev. Food Sci. Nutr..

[95] Benalaya I., Alves G., Lopes J., Silva L.R. (2024). A Review of Natural Polysaccharides: Sources, Characteristics, Properties, Food, and Pharmaceutical Applications. Int. J. Mol. Sci..

[96] Meng Y., Qiu C., Li X., McClements D.J., Sang S., Jiao A., Jin Z. (2024). Polysaccharide-based nano-delivery systems for encapsulation, delivery, and pH-responsive release of bioactive ingredients. Crit. Rev. Food Sci. Nutr..

[97] Kumari S., Debbarma R., Hussain S. (2025). Encapsulation strategies for enhancing the stability and shelf life of citrus bioactive compounds. Eur. Food Res. Technol..

[98] de Oliveira-Filho R.D., e Silva A.R.A., Moreira R.A., Nogueira N.A.P. (2018). Biological activities and pharmacological applications of cyclodextrins complexed with essential oils and their volatile components: A systematic review. Curr. Pharm. Des..

[99] Casalini S., Giacinti Baschetti M. (2023). The use of essential oils in chitosan or cellulose-based materials for the production of active food packaging solutions: A review. J. Sci. Food Agric..

[100] Yiasmin M.N., Al Azad S., Easdani M., Islam M.S., Hussain M., Cao W., Chen N., Uriho A., Asaduzzaman M., Liu C. (2025). The state-of-the-art on exploring polysaccharide–protein interactions and its mechanisms, stability, and their role in food systems. Food Rev. Int..

[101] Zambito Y., Piras A.M., Fabiano A. (2022). Bergamot Essential Oil: A Method for Introducing It in Solid Dosage Forms. Foods.

[102] Liu T., Zhao C., Chen Y., Wang X., Xiao H., Zheng J. (2025). Advances in efficient encapsulation and controlled release strategies for citrus oils. Crit. Rev. Food Sci. Nutr..

[103] Ajeeshkumar K.K., Aneesh P.A., Raju N., Suseela M., Ravishankar C.N., Benjakul S. (2021). Advancements in liposome technology: Preparation techniques and applications in food, functional foods, and bioactive delivery: A review. Compr. Rev. Food Sci. Food Saf..

[104] Xiao Y., Ahmad T., Belwal T., Aadil R.M., Siddique M., Pang L., Xu Y. (2023). A review on protein based nanocarriers for polyphenols: Interaction and stabilization mechanisms. Food Innov. Adv..

[105] Rasheed H.A., Rehman A., Li C., Bai M., Karim A., Dai J., Cui H., Lin L. (2024). Fabrication of *Citrus bergamia* essential oil-loaded sodium caseinate/peach gum nanocomplexes: Physicochemical, spectral, and structural characterization. Int. J. Biol. Macromol..

[106] Zhang L., Zhang M., Ju R., Mujumdar A.S., Deng D. (2024). Recent advances in essential oil complex coacervation by efficient physical field technology: A review of enhancing efficient and quality attributes. Crit. Rev. Food Sci. Nutr..

[107] Luque-Alcaraz A.G., Velazquez-Antillón M., Hernández-Téllez C.N., Graciano-Verdugo A.Z., García-Flores N., Iriqui-Razcón J.L., Silvas-García M.I., Zazueta-Raynaud A., Moreno-Vásquez M.J., Hernández-Abril P.A. (2022). Antioxidant Effect of Nanoparticles Composed of Zein and Orange (*Citrus sinensis*) Extract Obtained by Ultrasound-Assisted Extraction. Materials.

[108] Özdemir K.S., Azarabadi N., Topuz A. (2018). Microencapsulation of bergamot peel essential oil with gum arabic and maltodextrin blends: Stability and release characteristics of the essential oil compounds. Gıda.

[109] Chew S.C., Tan C.P., Nyam K.L. (2018). Effect of gum Arabic, β-cyclodextrin, and sodium caseinate as encapsulating agent on the oxidative stability and bioactive compounds of spray-dried kenaf seed oil. J. Food Sci..

[110] Nguyen M.M., Karboune S. (2023). Combinatorial Interactions of Essential Oils Enriched with Individual Polyphenols, Polyphenol Mixes, and Plant Extracts: Multi-Antioxidant Systems. Antioxidants.

[111] Liu T., Chen Y., Zhao S., Guo J., Wang Y., Feng L., Shan Y., Zheng J. (2023). The sustained-release mechanism of citrus essential oil from cyclodextrin/cellulose-based Pickering emulsions. Food Hydroc..

[112] Bartella L., Mazzotti F., Talarico I.R., De Luca G., Santoro I., Prejanò M., Riccioni C., Marino T., Di Donna L. (2022). Structural Characterization of Peripolin and Study of Antioxidant Activity of HMG Flavonoids from Bergamot Fruit. Antioxidants.

[113] Custureri I.M.G., Giuffrè A.M., Loizzo M.R., Tundis R., Soria A.C., Sicari V. (2024). Bergamot flavoured olive oil: Comparison between enrichment processes, evaluation of shelf-life and health properties. Appl. Food Res..

[114] Multari S., Carlin S., Sicari V., Martens S. (2020). Differences in the composition of phenolic compounds, carotenoids, and volatiles between juice and pomace of four citrus fruits from Southern Italy. Eur. Food Res. Technol..

[115] Trovato E., Arigò A., Vento F., Micalizzi G., Dugo P., Mondello L. (2021). Influence of Citrus Flavor Addition in Brewing Process: Characterization of the Volatile and Non-Volatile Profile to Prevent Frauds and Adulterations. Separations.

[116] Trovato E., Russo M., Cucinotta L., Oulad El Majdoub Y., Testa Camillo M.R., De Grazia G., Arigò A., Sciarrone D., Mondello L., Dugo P. (2023). Quality evaluation of flavoured extra-virgin olive oils according to their chemical composition. Food Anal. Methods.

[117] Mollace R., Macrì R., Nicita M., Musolino V., Gliozzi M., Carresi C., Bava I., Maiuolo J., Tavernese A., Cardamone A. (2023). Bergamot Polyphenolic Extract Combined with Albedo and Pulp Fibres Counteracts Changes in Gut Microbiota Associated with High-Fat Diet: Implications for Lipoprotein Size Re-Arrangement. Int. J. Mol. Sci..

[118] Lertnirundon N., Mahidsanan T. (2020). Effectiveness of bergamot juice on the survival of Bacillus cereus and quality of Thai steamed pumpkin cake. Food Appl. Biosci. J..

[119] Carabetta S., Di Sanzo R., Andronaco P., Canino F., Branyik T., Salafia F., Fuda S., Muscolo A., Russo M. (2024). UHPLC-PAD Protocol for the Simultaneous Identification of Polyphenols and Bitter Acids: Organoleptic and Nutraceutical Fingerprinting of Bergamot-Flavored Craft Beer. Foods.

[120] Barreca D., Mandalari G., Calderaro A., Smeriglio A., Trombetta D., Felice M.R., Gattuso G. (2020). Citrus Flavones: An Update on Sources, Biological Functions, and Health Promoting Properties. Plants.

[121] Zappia A., Branca M.L., Piscopo A., Poiana M. (2020). Shelf life extension of mozzarella cheese packed in preserving liquid with calcium lactate and bergamot juice concentrate. J. Dairy. Res..

[122] Palumbo M., Quintieri L., Bartella L., Di Donna L., Santoro I., Luparelli A., Caputo L., Pace B., Cozzolino R., Matarazzo C. (2026). Improvement of antioxidant activity and postharvest quality of fresh-cut artichoke by dipping in flavonoid-rich bergamot juice. Postharvest Biol. Technol..

[123] Demircan B., Velioglu Y.S. (2024). The effect of phenolics from bergamot juice powder extracted with different solvents on the physicochemical and antibacterial properties of chitosan films. Food Packag. Shelf Life.

[124] Demircan B., Velioglu Y.S. (2024). Control of Browning, Enzyme Activity, and Quality in Stored Fresh-cut Fruit Salads through Chitosan Coating Enriched with Bergamot Juice Powder. Foods.

[125] De Bruno A., Gattuso A., Ritorto D., Piscopo A., Poiana M. (2023). Effect of Edible Coating Enriched with Natural Antioxidant Extract and Bergamot Essential Oil on the Shelf Life of Strawberries. Foods.

[126] Arigò A., Rigano F., Russo M., Trovato E., Dugo P., Mondello L. (2021). Dietary Intake of Coumarins and Furocoumarins through Citrus Beverages: A Detailed Estimation by a HPLC-MS/MS Method Combined with the Linear Retention Index System. Foods.

[127] Hashemi S.M.B., Jafarpour D. (2020). Fermentation of bergamot juice with Lactobacillus plantarum strains in pure and mixed fermentations: Chemical composition, antioxidant activity and sensorial properties. LWT.

[128] Algieri C., Bernardini C., Oppedisano F., La Mantia D., Trombetti F., Palma E., Forni M., Mollace V., Romeo G., Nesci S. (2022). Mitochondria Bioenergetic Functions and Cell Metabolism Are Modulated by the Bergamot Polyphenolic Fraction. Cells.

[129] Cruzeiro J., Belin M.A.F., Sormani L.E., Vieira T.A., Grandini N.A., Sforca M., Moreto F. (2025). Effect of bergamot leaf extract (*Citrus bergamia*) on metabolic, lipid, and oxidative imbalance in the liver of obese rats. Food Res. Int..

[130] Trombetta D., Cimino F., Cristani M., Mandalari G., Saija A., Ginestra G., Bisignano G., Saija A. (2010). In Vitro Protective Effects of Two Extracts from Bergamot Peels on Human Endothelial Cells Exposed to Tumor Necrosis Factor-α. J. Agric. Food Chem..

[131] Lamiquiz-Moneo I., Giné-González J., Alisente S., Bea A.M., Pérez-Calahorra S., Marco-Benedí V., Baila-Rueda L., Jarauta E., Cenarro A., Civeira F. (2020). Effect of bergamot on lipid profile in humans: A systematic review. Crit. Rev. Food Sci. Nutr..

[132] Sadeghi-Dehsahraei H., Esmaeili Gouvarchin Ghaleh H., Mirnejad R., Parastouei K. (2022). The effect of bergamot (KoksalGarry) supplementation on lipid profiles: A systematic review and meta-analysis of randomized controlled trials. Phytother. Res..

[133] Rondanelli M., Peroni G., Riva A., Petrangolini G., Allegrini P., Fazia T., Bernardinelli L., Naso M., Faliva M.A., Tartara A. (2021). Bergamot phytosome improved visceral fat and plasma lipid profiles in overweight and obese class I subject with mild hypercholesterolemia: A randomized placebo controlled trial. Phytother. Res..

[134] Fogacci F., Giovannini M., Di Micoli A., Fiorini G., Grandi E., Borghi C., Cicero A.F.G. (2024). A Randomized, Double-Blind, Placebo-Controlled Clinical Trial on the Effect of a Dietary Supplement Containing Dry Artichoke and Bergamot Extracts on Metabolic and Vascular Risk Factors in Individuals with Suboptimal Cholesterol Levels. Nutrients.

[135] Minoretti P., Lista S., Khoramipour K., Crespo-Escobar P., Santos-Lozano A., Emanuele E. (2025). Hypolipidemic, anti-inflammatory, and neurotrophic effects of a multicomponent nutraceutical containing berberine, bergamot, and amaranth in mild-to-moderate hypercholesterolemia. Neuro Endocrinol. Lett..

[136] Russo B., Picconi F., Malandrucco I., Frontoni S. (2019). Flavonoids and Insulin-Resistance: From Molecular Evidences to Clinical Trials. Int. J. Mol. Sci..

[137] Fogacci F., Giovannini M., Imbalzano E., Grandi E., Rizzoli E., D’Addato S., Cicero A.F.G. (2023). Metabolic and vascular effect of a new standardized bergamot phytocomplex: A three-arm, placebo-controlled, double-blind clinical trial. Arch. Med. Sci..

[138] Remigante A., Spinelli S., Straface E., Gambardella L., Russo M., Cafeo G., Caruso D., Falliti G., Dugo P., Dossena S. (2023). Mechanisms underlying the anti-aging activity of bergamot (*Citrus bergamia*) extract in human red blood cells. Front. Physiol..

[139] Ferlazzo N., Cirmi S., Maugeri A., Russo C., Lombardo G.E., Gangemi S., Calapai G., Mollace V., Navarra M. (2020). Neuroprotective Effect of Bergamot Juice in 6-OHDA-Induced SH-SY5Y Cell Death, an In Vitro Model of Parkinson’s Disease. Pharmaceutics.

[140] Ilari S., Lauro F., Giancotti L.A., Malafoglia V., Dagostino C., Gliozzi M., Condemi A., Maiuolo J., Oppedisano F., Palma E. (2021). The Protective Effect of Bergamot Polyphenolic Fraction (BPF) on Chemotherapy-Induced Neuropathic Pain. Pharmaceuticals.

[141] Barbarossa A., Mallamaci R., Spinozzi E., Maggi F., Sgobba M.N., Rosato A., Carocci A., Meleleo D. (2025). Investigating Bergamot Essential Oil (BEO) Properties: Cytoprotection in Neuronal Cells Exposed to Heavy Metals and Antibacterial Activities. Antioxidants.

[142] Parafati M., La Russa D., Lascala A., Crupi F., Riillo C., Fotschki B., Mollace V., Janda E. (2024). Dramatic Suppression of Lipogenesis and No Increase in Beta-Oxidation Gene Expression Are among the Key Effects of Bergamot Flavonoids in Fatty Liver Disease. Antioxidants.

[143] Mazzola G., Rondanelli M., Baron G., Zupo R., Castellana F., Clodoveo M.L., Gasparri C., Barrile G.C., Seniga M., Schiavi L.M. (2024). Bergamot (*Citrus bergamia*), a (Poly)Phenol-Rich Source for Improving Osteosarcopenic Obesity: A Systematic Review. Foods.

[144] Lascala A., Martino C., Ragusa S., Nucera S., Walker R., Gratteri S., Mollace V. (2016). Molecular mechanisms of lipid- and glucose-lowering activities of bergamot flavonoids. PharmaNutrition.

[145] Risitano R., Currò M., Cirmi S., Ferlazzo N., Campiglia P., Caccamo D., Ientile R., Navarra M. (2014). Flavonoid fraction of Bergamot juice reduces LPS-induced inflammatory response through SIRT1-mediated NF-κB inhibition in THP-1 monocytes. PLoS ONE.

[146] Maggiolo G., Aldigeri R., Savini C., Mengani M., Maggi M., Frigeri G., Spigoni V., Cinquegrani G., Fantuzzi F., Di Donna L. (2024). Chronic consumption of a bergamot-based beverage does not affect glucose, lipid and inflammatory biomarkers of cardiometabolic risk in healthy subjects: A randomised controlled intervention study. Food Funct..

[147] Jia Q., Zhu R., Tian Y., Chen B., Li R., Li L., Zhao D., Mo F., Cao Y., Qin Y. (2019). Salvia miltiorrhiza in Diabetes: A Review of Its Pharmacology, Phytochemistry, and Safety. Phytomedicine.

